# Colchicine Blocks Abdominal Aortic Aneurysm Development by Maintaining Vascular Smooth Muscle Cell Homeostasis

**DOI:** 10.7150/ijbs.93544

**Published:** 2024-03-17

**Authors:** Min Chen, Dafeng Yang, Yangzhao Zhou, Chongzhe Yang, Wenhui Lin, Jie Li, Jitao Liu, Jiamin Ye, Wenhui Huang, Wentao Ma, Wei Li, Jiyan Chen, Ying Zhang, Guo-Ping Shi, Jianfang Luo, Jie Li, Songyuan Luo

**Affiliations:** 1 Department of Cardiology, Guangdong Cardiovascular Institute, Guangdong Provincial Key Laboratory of Coronary Heart Disease Prevention, Guangdong Provincial People's Hospital, Southern Medical University, Guangzhou, China.; 2 Department of Cardiovascular Surgery, The Second Xiangya Hospital of Central South University, Changsha, Hunan, China.; 3 Department of Geriatrics, National Key Clinic Specialty, Guangzhou First People's Hospital, School of Medicine, South China University of Technology, Guangzhou, China.; 4 Department of Cardiology, Guangdong Provincial People's Hospital Zhuhai Hospital, Zhuhai, China.; 5 Department of Medicine, Brigham and Women's Hospital and Harvard Medical School, Boston, Massachusetts, USA.; 6 Department of Cardiology, Guangdong Cardiovascular Institute, Guangdong Provincial Key Laboratory of Hypertension, Guangdong Provincial People's Hospital, Southern Medical University, Guangzhou, China.; 7 Department of Cardiology, Ganzhou Hospital of Guangdong Provincial People's Hospital, Ganzhou Municipal Hospital, Ganzhou, Jiangxi, China.

**Keywords:** abdominal aortic aneurysm, colchicine, vascular smooth muscle cell, sclerostin, N6-methyladenosine

## Abstract

Development of non-surgical treatment of human abdominal aortic aneurysm (AAA) has clinical significance. Colchicine emerges as an effective therapeutic regimen in cardiovascular diseases. Yet, whether colchicine slows AAA growth remain controversy. Here, we demonstrated that daily intragastric administration of low-dose colchicine blocked AAA formation, prevented vascular smooth muscle cell (SMC) phenotype switching and apoptosis, and vascular inflammation in both peri-aortic CaPO_4_ injury and subcutaneous angiotensin-II infusion induced experimental AAA mice models. Mechanistically, colchicine increased global mRNA stability by inhibiting the METTL14/YTHDC1-mediated m6A modification, resulting in increased sclerostin (SOST) expression and consequent inactivation of the WNT/β-catenin signaling pathway in vascular SMCs from mouse AAA lesions and in cultured human aortic SMCs. Moreover, human and mouse AAA lesions all showed increased m6A methylation, decreased SOST expression, and skewed synthetic SMC de-differentiation phenotype, compared to those without AAA. This study uncovers a novel mechanism of colchicine in slowing AAA development by using the METTL14/SOST/WNT/β-catenin axis to control vascular SMC homeostasis in mouse aortic vessels and in human aortic SMCs. Therefore, use of colchicine may benefit AAA patients in clinical practice.

## Introduction

Abdominal aortic aneurysm (AAA) is one of the causes of sudden death in adults due to aortic rupture 1. At present, open surgical repair and endovascular intervention remain the only treatments 2. Therefore, considerable residual morbidity and mortality risks remain among these patients and there is no effective non-surgical medication available to prevent or slow AAA progression and rupture to date 2, 3. Exploring the underlying mechanisms and development of potential novel therapies are urgent.

The main pathological features of AAA include loss of contractile vascular smooth muscle cells (SMCs), aortic wall extracellular matrix (ECM) degradation, and immune cell infiltration and activation, all contribute to aortic wall remodeling throughout all stages of AAA development. Vascular SMCs are one of the major cell types located in the aortic medial layer. Quiescent SMCs are essential in maintaining aortic wall structural integrity and homeostasis 4. Yet, vascular SMCs exhibit remarkable plasticity upon different environmental stimuli termed phenotypic switching 5. Accumulating evidence demonstrates that vascular SMCs undergo phenotypic switching and exhibit reduced expression of contractile genes [e.g. smooth muscle actin (ACTA2), smooth muscle protein 22-alpha (SM22α, also called transgelin, TAGLN) and calponin (CNN1)], but enhanced expression of matrix-degrading protease (e.g. matrix metalloproteinases) and pro-inflammatory cytokines [e.g. tumor necrosis factor-α (TNF-α), interleukin-1β (IL-1β) and interleukin-6 (IL-6)], leading to vascular SMCs apoptosis, ECM degradation, and aortic wall inflammation and rupture 6-10. Inhibition or reverse of vascular SMC phenotypic switching holds great promise to develop pharmacological regimens to limit or prevent AAA development.

Colchicine is a well-known anti-inflammatory drug that is used commonly for the treatment of gout, pericarditis, familial Mediterranean fever, and Adamantiades-Behcet's syndrome 11. The anti-inflammatory action of colchicine was thought largely due to its property to bind to tubulin and depolymerize microtubules 12. However, the exact mechanism of how colchicine functions still remains incompletely understood. It is demonstrated that colchicine also selectively targets hepatocytes and induces production of anti-inflammatory hepatokines that subsequently inhibit myeloid cell activation and inflammation 13. Unexpectedly, colchicine was subsequently identified to emerge as an effective therapeutic agent in cardiovascular diseases and was approved by the U.S. Food and Drug Administration to use in adult patients with established atherosclerotic disease or with multiple risk factors for cardiovascular disease in June 2023 14-17. Colchicine may also provide similar protective effects on AAA 18, 19. Yet, other study yielded the opposite conclusion that colchicine did not reduced AAA growth 20. With these conflicting results, whether colchicine affects AAA development remains unknown. In the present study, we showed that low-dose of colchicine inhibited peri-aortic CaPO_4_ injury- and angiotensin-II (Ang-II) infusion-induced AAA in mice. Moreover, we identified a novel mechanism that colchicine slowed AAA development by inactivating the WNT/β-catenin pathway in a methyltransferase-like 14 (METTL14)/YTH domain containing-1 (YTHDC1)/sclerostin (SOST)-dependent manner to maintain vascular SMC homeostasis and prevent vascular inflammation. This work suggests that colchicine serves as a potential therapeutic regimen for AAA.

## Materials and Methods

### Animal studies

Male 8 to 10 weeks old apolipoprotein E-deficient (*Apoe^-/-^*) mice (C57BL/6J background) and wild-type (WT, C57BL/6J background) mice were purchased from Hunan SJA Laboratory Animal Co. in China. All mice were housed under a 12-hour light/dark cycle in a pathogen-free animal facility with free access to food and water. Subcutaneous angiotensin-II (Ang-II) infusion- and peri-aortic CaPO_4_ injury-induced AAA was performed as described previously 21, 22. Briefly, mice were anaesthetized with ketamine (100 mg/kg) and pentobarbital sodium (50 mg/kg). After anesthesia, *Apoe^-/-^* mice were subcutaneously infused with Ang-II (1000 ng/kg/min, A9525, Sigma-Aldrich, St. Louis, MO) delivered by osmotic minipumps (Model 2004, Alzet, Durect Corporation, Cupertino, CA) and kept on a 1.25% high cholesterol diet (D12108C, Research Diets, New Brunswick, NJ) for 28 days. Body weight and blood pressures were recorded before surgery and 28 days after surgery. For peri-aortic CaPO_4_ injury-induced AAA, the abdominal aorta of C57BL/6J mice between renal arteries and bifurcation of the iliac arteries was carefully isolated and several cotton balls soaked in 0.5 M CaCl_2_ were applied around the aorta for 10 min and replaced by several PBS-soaked cotton balls for another 5 min, and then kept on a standard chow diet for 7 days. To knock down the SOST expression in the local abdominal aorta, 20 nmol of SOST small interfering RNA (siRNA) (SiBDMV002-105, RiboBio, China) or non-specific control siRNA (siN0000001-1-5, RiboBio, China) was dissolved in 50 μl Dulbecco's phosphate-buffered saline (dPBS, solution 1). Lipofectamine 2000 (25 μl, 11668019, Invitrogen, Waltham, MA) was mixed with 25 μl dPBS by pipetting up and down (solution 2), and placed at room temperature for 15 min. Solution 1 and 2 were then mixed by pipetting up and down and incubated at room temperature for 30 min. After incubation, the mixture was dissolved in Pluronic gel (30%, P2443, Sigma-Aldrich) on ice and placed surrounding the CaPO_4_-injured abdominal aorta. After surgery, mice received daily intragastric administration of colchicine (0.2 mg/kg, C3915, Sigma-Aldrich) dissolved in saline according to prior studies 13 or the same volume of saline. After 7 days or 28 days of AAA induction, mice were sacrificed with carbon dioxide narcosis and the abdominal aorta was carefully isolated and photographed. Aortic diameters were measured. All animal procedures conformed to the Guide for the Care and Use of Laboratory Animals published by the US National Institutes of Health and was approved by the Guangdong Provincial People's Hospital Standing Committee on Animals.

### Cell culture and transfection

Human aortic SMCs were purchased from Meisen CTCC (CTCC-170-HUM, China) and cultured in SMC growth medium SMCM (1101, ScienCell, USA). Cells were cultured in a humidified atmosphere of 95% air and 5% CO2 at 37 °C, and passages less than five were used for all experiments. Human aortic SMCs were plated 40,000/well on a 12-well plate, 70,000/well on a 6-well plate, or 150,000/well on a 10-cm dish for RNA extraction, protein preparation, or immunofluorescent staining. Cells were grown to 80-90% confluence and treated with colchicine (1 nM, C3915, Sigma-Aldrich) and 20 ng/ml platelet-derived growth factor BB (PDGF-BB, HZ-1308, Proteintech Group, Rosemont, IL), or colchicine with PDGF-BB and WNT pathway agonist WNT agonist 1 (10 μM, S8178, Selleck Chemicals, Houston, TX) for 24 hours and then harvested for indicated experiments. For SOST and METTL14 loss-of-function experiments, 100 nM siRNA specific for SOST (M518200-9000, Sangon Biotech, China), METTL14 (M518200-9002, Sangon Biotech, China), YTHDC1 (M518200-9003, Sangon Biotech), or non-specific control siRNA (R19837, Sangon Biotech) were transfected using lipofectamine 2000 (11668019, Invitrogen) according to the manufacture's instruction. After 24 hours transfection, cells were treated with 1 nM colchicine and 20 ng/ml PDGF-BB or colchicine with 20 ng/ml PDGF-BB and 10 μM WNT agonist 1 for 24 hours and then harvested for indicated experiments.

### Cytoplasmic and nuclear protein extraction

Cytoplasmic and nuclear proteins from human aortic SMCs and AAA lesions were isolated using a nuclear and cytoplasmic protein extraction kit (P0027, Beyotime, China) according to the manufacturer's instructions. Proteins were then processed for further immunoblot assay.

### Dot blot assay

Total mRNA (250 ng, 500 ng or 1000 ng) isolated from AAA lesions or human aortic SMCs was denatured at 95 °C for 3 min, followed by cooling down on ice immediately. Denatured mRNA dilutions were spotted on a BrightStar^@^ plus positively charged Nylon membrane (AM10102, Thermo Fisher Scientific, Waltham, MA) using Bio-Dot Microfiltration System (Bio-Rad Laboratories, Hercules, CA) according to the manufacturer's instructions. The membrane was cross-linked under UV light and blocked with 5% BSA in PBS-Tween for 30 min at room temperature, followed by incubation with anti-m6A antibody (1:1000, 202003, Synaptic Systems, Göttingen, Germany) overnight at 4 °C. Membrane was then incubated with secondary antibody at room temperature for 1 hour. Immunoblots were developed using a chemiluminescent reagent (P0018M, Beyotime). The same amount of mRNAs were spotted on the membrane, stained with 0.02% methylene blue in 0.3 M sodium acetate (pH 5.2) for 2 hours and washed with RNase-free water for 1 hour. Blot intensity was quantified using the Image J software.

### ELISA analysis

Plasma levels of total cholesterol (TCHO, A111-1-1), triglyceride (TG, A110-1-1), low-density lipoprotein (LDL-c, A113-1-1) and high-density lipoprotein cholesterol (HDL-c, A112-1-1), aspartate aminotransferase (AST, C010-2-1), alanine aminotransferase (ALT, C009-2-1) and creatinine (Cr, C011-2-1) were measured using commercial colorimetric enzymatic assay kit from Nanjing Jiancheng Bioengineering according to the manufacturer's instruction. Plasma TNF-α (RK00027, ABclonal, Woburn, MA), IL-1β (RK00006, ABclonal), IL-6 (Rk00008, ABclonal) and NETs (JL47089, Jonlon Biotech, Wuhan, China) levels were quantitated using relevant ELISA kits according to the manufacturer's instructions. Human (E-EL-H1544c) and mouse (E-EL-M2435c) plasma SOST levels, as well as human (E-EL-H0080c) and mouse (E-EL-M0604c) plasma GDF 15 levels, were measured using ELISA Kit from Elabscience (Houston, TX) following the manufacturer's instructions.

### Immunohistochemical and immunofluorescent staining

Immunohistochemical and immunofluorescent staining were performed as described previously 21-23. Briefly, serial cryostat sections (6 μm) from mice or paraffin sections (6 μm) from human AAA lesion were prepared and processed for immunostaining to detect α-smooth muscle actin (α-SMA, 1:100, A17910, ABclonal), Mac2 (1:100, CL8942AP, Cedarlane, Burlington, Canada) and CD31 (1:250, ab7388, Abcam, Cambridge, MA). For immunofluorescent staining, sections were stained with FITC labeled anti-α-SMA (1:500, F3777, Sigma-Aldrich), α-tubulin (1:200, 11224-1-AP, Proteintech Group), β-catenin (1:200, 8814S, Cell Signaling Technology, Danvers, MA), CD68 (1:200, ab955, Abcam), KLF4 (Krüppel-like factor 4, 1:100, A13673, ABclonal), Ly6G (1:250, BP0075-1, BioXCell, Lebanon, NH), m6A (1:100, 202003, Synaptic Systems), METTL14 (1:200, A8530, ABclonal), SOST (1:200, 21933-1-AP, Proteintech Group), TAGLN (1:200, ab14106, Abcam), VCAM-1 (1:250, ab134047, Abcam), or CD31 (1:250, ab7388, Abcam). Lesion cell proliferation was detected by immunofluorescent staining with Ki67 (1:200, ab15580, Abcam) and FITC labeled anti-α-SMA (1:500, F3777, Sigma-Aldrich). Lesion cell apoptosis was measured by immunofluorescent staining with TUNEL (C1090, Beyotime Biotechnology, Shanghai, China) and FITC labeled anti-α-SMA (1:500, F3777, Sigma-Aldrich). Secondary antibodies used Alexa Fluor 488 (1:300, A11029 or A11006, Thermo Fisher Scientific) or Alexa Fluor 555 (1:300, A21428 or A21432, Thermo Fisher Scientific). The nuclei were stained with DAPI (R37606, Thermo Fisher Scientific). Immunohistochemical images were captured by Nikon Ti2 microscopy (Nikon, Tokyo, Japan) and immunofluorescent images were captured by Nikon A1 confocal laser scanning microscopy (Nikon). CD31-positive microvessels and Ly6G-positive neutrophils were counted manually and presented as numbers per aortic section. The Mac2-postive macrophage and α-SMA-positive SMC contents within AAA lesion were determined by detecting the staining intensity with computer-assisted image analysis software (Image-Pro Plus; Media Cybernetics, Bethesda, MD) and data were presented as a ratio of positive area to the tissue area. The β-catenin and α-SMA double positive SMCs, TUNEL and α-SMA double positive cells, Ki67 and α-SMA double positive cells, KLF4 and α-SMA double positive cells, SOST and α-SMA double positive cells, and CD68 and α-SMA double positive cells, and mean intensity of VCAM-1 and KLF4 in endothelial cells (ECs) were determined by detecting the staining intensity with Image-Pro Plus and data were presented as a ratio of positive area to media area or adventitial area.

To assess elastica fragmentation in AAA lesions, a commercial Verhoeff Van Gieson (EVG) staining kit (ab150667, Abcam) was used according to the manufacturer's instructions. The grade of elastin fragmentation was graded as previously described 24. Each aortic vessel was independently evaluated by two researchers.

### Methylated RNA immunoprecipitation (MeRIP) qPCR assay

To detect m6A modifications of SOST transcript, total RNA was extracted from normal abdominal aorta, AAA lesion, or human aortic SMCs and MeRIP was performed using a commercial Magna methylated RNA immunoprecipitation (MeRIP) m6A kit (17-10499, Millipore, Burlington, MA) according to the manufacturer's instructions. Briefly, 30 μg of total RNA was mixed with fragmentation buffer and then incubated at 94 ºC for 4 min followed by adding 2 μl of 0.5 M EDTA. Fragment RNA was further purified using RNase MiniElute Kit (74204, Qiagen, Hilden, Germany) according to the manufacturer's instructions. Fragmented total RNA was incubated with 5 μg of rabbit IgG (3000-0-AP, Proteintech Group) or anti-m6A antibodies (202003, Synaptic Systems) for immunoprecipitation. m6A methylation-enriched SOST mRNA was measured using real-time qPCR (RT-PCR) analysis. The primers were listed in **Table S1**.

### mRNA stability assay

mRNA stability assay was performed as described previously 23. Briefly, human aortic SMCs were stimulated with 20 ng/ml PDGF-BB plus 5 μg/ml actinomycin D (50760, Sigma-Aldrich) with or without 1 nM colchicine for indicated time points. Then cells were harvested, and total RNA was extracted and analyzed by RT-PCR. The remaining mRNA was determined by comparison with the expression level of the relevant gene at zero-time point (designated 100%) when actinomycin D was added.

### Immunobot analysis

AAA tissues were homogenized using TissueLyser II (Qiagen) according to the manufacturer's instruction. Total proteins from homogenized AAA tissue and cultured human aortic SMCs were extracted using RIPA lysis buffer (KGP702-100, KeyGen Biotech, China) with PMSF (Cat# GC10477, GLPBIO, Montclair, CA) and phosphatase inhibitor cocktail (GK10012, GLPBIO). The protein concentrations were measured using a BCA assay kit (KGP902, KeyGen Biotech) and then separated by 10% SDS-PAGE gels, transferred to PVDF membranes (IPVH00010, Millipore) and blocked in 5% non-fat milk for 1 hour at room temperature. Membranes were then incubated with the relevant antibodies including rabbit anti-METTL14 (1:1000, A8530, ABclonal), rabbit anti-METTL 3 (1:1000, A8370, ABclonal), rabbit anti-METTL 4 (1:1000, A9294, ABclonal), rabbit anti-α-SMA (1:1000, A17910, ABclonal), rabbit anti-KLF4 (1:1000, A13673, ABclonal), mouse anti-CD68 (1:1000, ab955, Abcam), rabbit anti-TAGLN (1:1000, ab14106, Abcam), rabbit anti-SOST (1:1000, MA523897, Thermo Fisher Scientific), rabbit anti-β-Catenin (1:1000, 8814S, Cell Signaling Technology), rabbit anti-glycogen synthase kinase-3β (GSK-3β, 1:1000, 12456S, Cell Signaling Technology), rabbit anti-P-GSK-3β (1:1000, 5558S, Cell Signaling Technology), mouse anti-GAPDH (1:5000, AC033, ABclonal) or rabbit anti-β-actin (1:4000, AC026, ABclonal) overnight at 4 ºC. Immunoblots were developed using a chemiluminescent reagent (P0018M, Beyotime) according to the manufacturer's instructions.

### *In situ* zymography

AAA lesion matrix metalloproteinase (MMP) activities were determined using a commercial kit (RTD6143, RealTimes, China) followed by the manufacturer's instructions. Briefly, 20 μg homogenized abdominal aortic tissue lysate was subjected to a 10% SDS-PAGE gel containing 0.1% gelatin (G9136, Sigma-Aldrich). The gel was then incubated in 2.5% Triton X-100 for 90 min to remove SDS, followed by incubation in a developing buffer overnight. Gel was then incubated in a staining buffer containing 0.1% Coomassie blue R-250 (1610400, Bio-Rad laboratories), 30% methanol, and 10% glacial acetic acid, and de-stained for 2 hours in a de-staining buffer containing 30% methanol and 10% acetic acid.

### RNA-Sequencing analysis

TRIzol reagent (AG21102, Accurate Biology) was used for total RNA isolation from AAA lesions according to the manufacturer's instructions. RNA-Sequencing analysis was performed as described previously 23, 25. Briefly, a standard mRNA library was generated and sequenced by Novogene Technology (Beijing, China). All sequencing experiments were run on an Illumina NovaSeq sequencer (Illumina, San Diego, CA). Differential expression analysis at the gene level was performed using the DESeq2 R package (1.20.0). Genes with FDR < 0.05 and log_2_fold-change > 1 were defined as differentially expressed genes for each comparison. DAVID tool was used to conduct Gene Ontology (GO) pathway enrichment analysis. Gene set enrichment analysis (GSEA) was applied to identify the significantly enriched pathways between the two groups using the GSEA 4.3.2 desktop application.

### RNA immunoprecipitation (RIP)

RIP was performed as described previously 25. Briefly, whole cell lysate was prepared by resuspending 10^6^ human aortic SMC pellet in 1 ml ice-cold RIP buffer (150 mM KCl, 25 mM Tris-HCI pH7.4, 5 mM EDTA, 0.5% NP-40, 0.5 mM 1,4-dithiothreitol) containing 100 units of RNase (R0102, Beyotime) and protease inhibitor cocktail (1 x final concentration, BL612A, Biosharp, China) and homogenized by 18 strokes using a dounce homogenizer, followed by centrifugation for 15 min at 15,000 g. The supernatant was cleared by applying 40 μl of magnetic protein A/G beads (HY-K0202, MedChemExpress USA, Monmouth Junction, NJ) and 5 μg normal control rabbit IgG (3000-0-AP, Proteintech Group). After taking 50 μl lysate as input control, the remaining lysate is divided into multiple portions of equal volume for 2 µg antibodies against YTHDC1 (ab264375, Abcam), YTHDC2 (35440S, Cell Signaling Technology), YTHDF1 (17479-1-AP, Proteintech Group), YTHDF2 (24744-1-AP, Proteintech Group), YTHDF3 (A8395, Abconal), and control rabbit IgG (3000-0-AP, Proteintech Group). The lysates were incubated with antibodies overnight at 4 ^o^C. The RNA-protein complexes were extracted by adding 40 μl of magnetic protein A/G beads and incubated for 1 hour at 4 ^o^C. Then, beads were washed three times with ice-cold RIP buffer and the RNA was extracted using TRIzol reagent. Subsequent RT-PCR was performed using the same input volume.

### RNA extraction and real-time qPCR

AAA lesions were homogenized using TissueLyser II (Qiagen) according to the manufacturer's instructions. Total RNA from homogenized AAA lesions or human aortic SMCs was isolated using TRIzol reagent (AG21102, Accurate Biology, China) according to manufacturer's protocol and quantified spectrophotometrically using Nanodrop 2000 (Thermo Fisher Scientific). A reverse transcription kit (AG11707, Accurate Biology) was used to generate the cDNA, and SYBR® Green Pro Taq HS Premix Mix (AG11701, Accurate Biology) was used for RT-PCR. Expression of the relative mRNA levels were normalized to human GAPDH or mouse β-actin. All primers are listed in **Table S2**.

### Patient sample collection

The use of discarded and de-identified human aortic specimens or blood samples (protocol No. GDREC2018215H-R3) was approved by the Institution Research Ethics Committee of Guangdong Provincial People's Hospital and conducted under the guidance of the Declaration of Helsinki. Informed consent was obtained from each patient before collection of samples. Abdominal aortic samples were obtained from 6 AAA patients and 3 healthy organ donors during surgery. After aortic samples were collected, formalin fixation and paraffin embedding were performed using standard methods. De-identified plasma samples were prepared from blood collection in EDTA-coated tubes from 36 AAA patients within 24 hours of admission and 36 age- and sex-matched control subjects without AAA. The plasma sample was isolated from whole blood at 1,500 g for 15 min at room temperature and stored at -80 ºC.

### Statistical analysis

GraphPad Prism software (Version 8.4, GraphPad Software, Inc, Boston, MA) or RStudio (for RNA-Seq) was used for statistical analyses. Unpaired 2-tailed Student's* t* test was used to examine the statistical significance between 2 groups with normally distributed continuous variables. For comparison of multiple groups, two-way ANOVA followed by Bonferroni post hoc test was used. Survival curves were described by Kaplan-Meier plots and compared with the long-rank test. For data without normal distribution, the nonparametric Mann-Whitney *U* test or χ^2^ test was used to compare 2 groups. All data are presented as mean ± SEM or median [interquartile range (IQR)] and *P* <0.05 was considered statistically significant.

## Results

### Colchicine inhibits mouse experimental AAA

**Figure 1:**
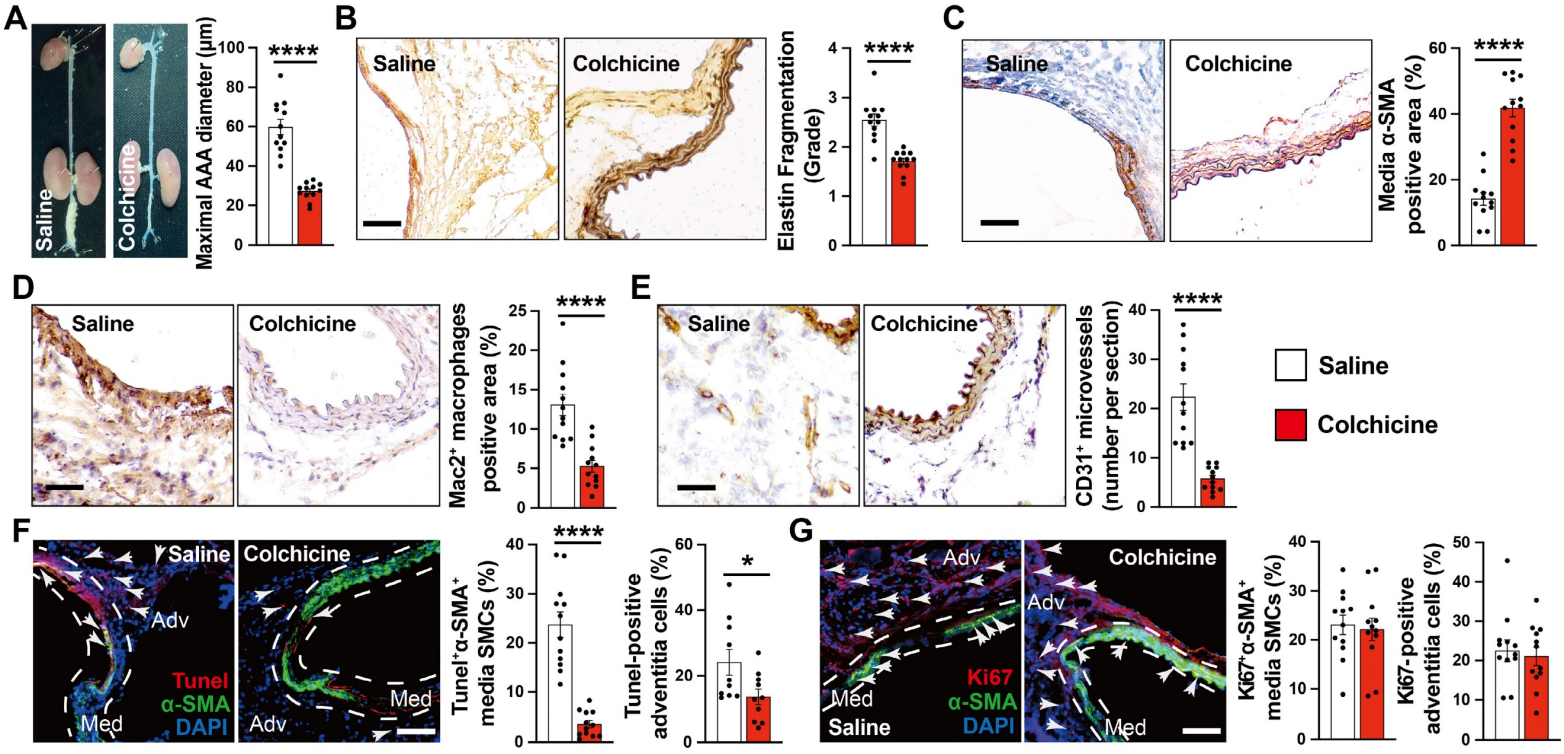
Colchicine prevents peri-aortic CaPO_4_ injury-induced AAA. **A)** Abdominal aortic diameters of saline and colchicine-treated mice.** B)** Elastin fragmentation grade.** C)** Lesion α-SMA^+^ SMCs positive area.** D)** Lesion Mac2^+^ macrophage-positive area.** E)** Lesion CD31^+^ microvessel numbers.** F)** Immunofluorescent staining of media and adventitia α-SMA (green) and TUNEL (red) double positive SMCs. Arrows indicate TUNEL-positive cells.** G)** Immunofluorescent staining of media and adventitia α-SMA (green) and Ki67 (red) double positive SMCs. Arrows indicate Ki67-positive cells. Representative images are shown to the left (**B-G**). Scale: 100 µm (**B-E**) and 200 µm (**F/G**). Data are mean ± SEM, n=12 per group. *P<0.05, ****P<0.0001, Student's* t* test.

To test whether colchicine has a protective role on AAA development, we produced peri-aortic CaPO_4_ injury-induced AAA in C57BL/6J mice and gavage mice either with colchicine (0.2 mg/kg/day) or the same volume of saline 13. After 7 days, we observed a significantly smaller abdominal aortic diameters and lesion sizes in mice that received colchicine than those received saline (**Fig. 1A**). Colchicine treatment significantly reduced AAA lesion media elastin fragmentation and media SMC loss (**Fig. 1B-C**). Histological analysis showed that AAA lesions from colchicine-treated mice contained fewer Mac2-positive macrophages and CD31-positive microvessels than those of saline-treated mice (**Fig. 1D-E**). Immunofluorescent double staining showed that colchicine significantly reduced lesion endothelial VCAM-1 expression, TUNEL^+^α-SMA^+^ double-positive apoptotic SMCs, but did not affect lesion Ki67^+^α-actin^+^ SMC proliferation (**Fig. 1F-G, and Fig. S2A**).

To confirm a protective role of colchicine on AAA formation, we also generated Ang-II infusion-induced AAA in *Apoe^-/-^* mice and gavage mice either colchicine or saline as we did to the peri-aortic CaPO_4_-injuried mice. At 28 days after Ang-II infusion, colchicine-treated *Apoe^-/-^* mice showed similar AAA incident rates to those of saline-treated mice (**Fig. S1A**). Yet, colchicine significantly reduced mouse mortality rate and these mice had much smaller suprarenal aortic diameters than those of saline-treated mice (**Fig. S1B-C**). Like in the CaPO_4_-injuried mice (**Fig. 1**), AAA lesions from colchicine-treated *Apoe^-/-^* mice also showed much less media elastica fragmentation, SMC loss and apoptosis, lesion macrophages accumulation, microvessel counts, and endothelial VCAM-1 expression, than those of saline-treated mice, but there were no differences in SMC proliferation between the groups (**Fig. S1 and Fig. S2C**). Also, there were no differences in body weight gain, or systolic and diastolic blood pressures before and after Ang-II perfusion between *Apoe^-/-^* mice treatment with colchicine or saline (**Fig. S3**). ELISA analysis showed that colchicine did not affect plasma lipid profiles (total cholesterol, triglyceride, LDL-c, and HDL-c) in mice with Ang-II-induced AAA (**Fig. S4A**), or liver and kidney functions (AST, ALT, creatinine) in mice with Ang-II infusion- or CaPO_4_ injury-induced AAA (**Fig. S4B and S4C**). These observations suggest that colchicine reduces aortic SMC loss, vascular inflammation, and angiogenesis and protects mice from AAA development independent of AAA models.

### Colchicine does not influence neutrophil activation but inhibits aortic SMC phenotypic switching during AAA development

**Figure 2:**
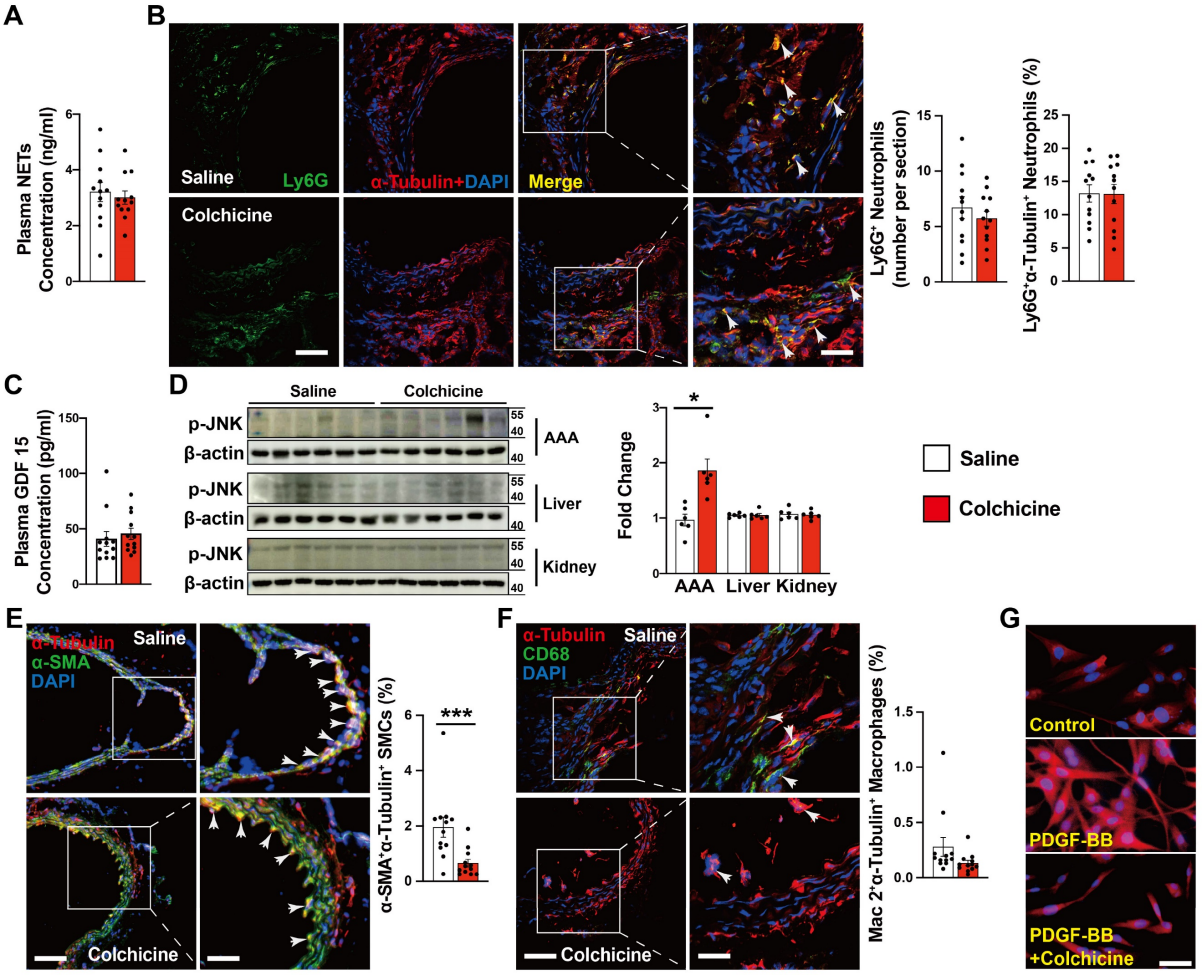
Colchicine does not influence AAA lesion neutrophil activation, but increases lesion SMC tubulin depolymerization in CaPO_4_ injury-induced AAA. **A)** ELISA analysis of plasma NET levels from saline and colchicine-treated mice. **B)** Immunofluorescent staining of Ly6G (green) and α-tubulin (red) to detect lesion neutrophil accumulation and tubulin depolymerization. Arrows indicate Ly6G-positive neutrophils. **C)** ELISA analysis of plasma GDF 15 levels from saline and colchicine-treated mice. **D)** Immunoblot analysis of JNK phosphorylation in AAA lesions, livers, and kidneys from saline and colchicine-treated mice. **E)** Immunofluorescent staining of α-SMA (green) and α-tubulin (red) to detect SMC tubulin depolymerization. Arrows indicate α-SMA-positive SMCs. **F)** Immunofluorescent staining of CD68 (green) and α-tubulin (red) to detect macrophage tubulin depolymerization. Arrows indicate CD68-positive macrophages. Representative images of **B**,** D**, **E**, and **F** are shown to the left. Scale: 100 μm, inset: 25 μm. **G)** Immunofluorescent staining of α-tubulin (red) to detect tubulin depolymerization in cultured human aortic SMCs stimulated with 20 ng/ml PDGF-BB and treatment with or without 1 nM colchicine (n=4). Scale: 50 µm. Data are mean ± SEM, n=12 per group. *P<0.05, ***P<0.001. Student's* t* test (**A, B,** and **D-F**) or nonparametric Mann-Whitney *U* test (**C**).

Neutrophils are probably the earliest inflammatory cells that accumulate in AAA lesions and contribute to AAA progression 21, 26. Colchicine preferentially targets neutrophils and affects neutrophil activity 12. It is possible that the protective role of colchicine on AAA may involve neutrophil activity inhibition. Yet, we did not detect any differences in plasma levels of neutrophil extracellular traps (NETs) or AAA lesion Ly6G-positive neutrophil accumulation from peri-aortic CaPO_4_-injured (**Fig. 2A-B**) and Ang-II-infused mice (**Fig. S5A-B**) with or without colchicine treatment, suggesting that low-dose of colchicine did not affect neutrophil activation or accumulation during AAA formation 13.

Previous studies indicated that colchicine distributes in a tissue-selective manner and accumulates mostly in liver and kidney. The anti-inflammatory actions of colchicine involve growth differentiation factor 15 (GDF15) secretion and hepatocyte-selective action 13, 27. To examine whether colchicine inhibits AAA formation also in a tissue-selective manner, we performed ELISA analysis of plasma samples from CaPO_4_ injured and Ang-II-infused mice and found that colchicine did not affect plasma GDF15 content (**Fig. 2C and Fig. S5C**). Because colchicine binds to tubulin and inhibits microtubule assembly, we performed immunoblot to examine the expression and phosphorylation of c-Jun N-terminal kinase (JNK), a microtubule depolymerization-induced cell stress biomarker 28. We found that colchicine treatment did not affect the p-JNK levels in the livers or kidneys, but significantly increased JNK phosphorylation in AAA lesions from colchicine-treated mice (**Fig. 2D**). Consistently, immunofluorescent double staining demonstrated that colchicine treatment reduced the tubulin levels in α-SMA-positive SMCs from AAA lesions from both CaPO_4_-injured and Ang-II-infused mice (**Fig. 2E, Fig. S5D**), but colchicine did not affect tubulin expression in Mac2-positive macrophages from these lesions (**Fig. 2F, Fig. S5E**). In cultured human aortic SMCs, colchicine treatment also reduced microtubule assembly, as indicated by reduced tubulin intensity (**Fig. 2G**). These observations suggest that colchicine affects the pathobiology of aortic SMCs in the context of AAA.

**Figure 3:**
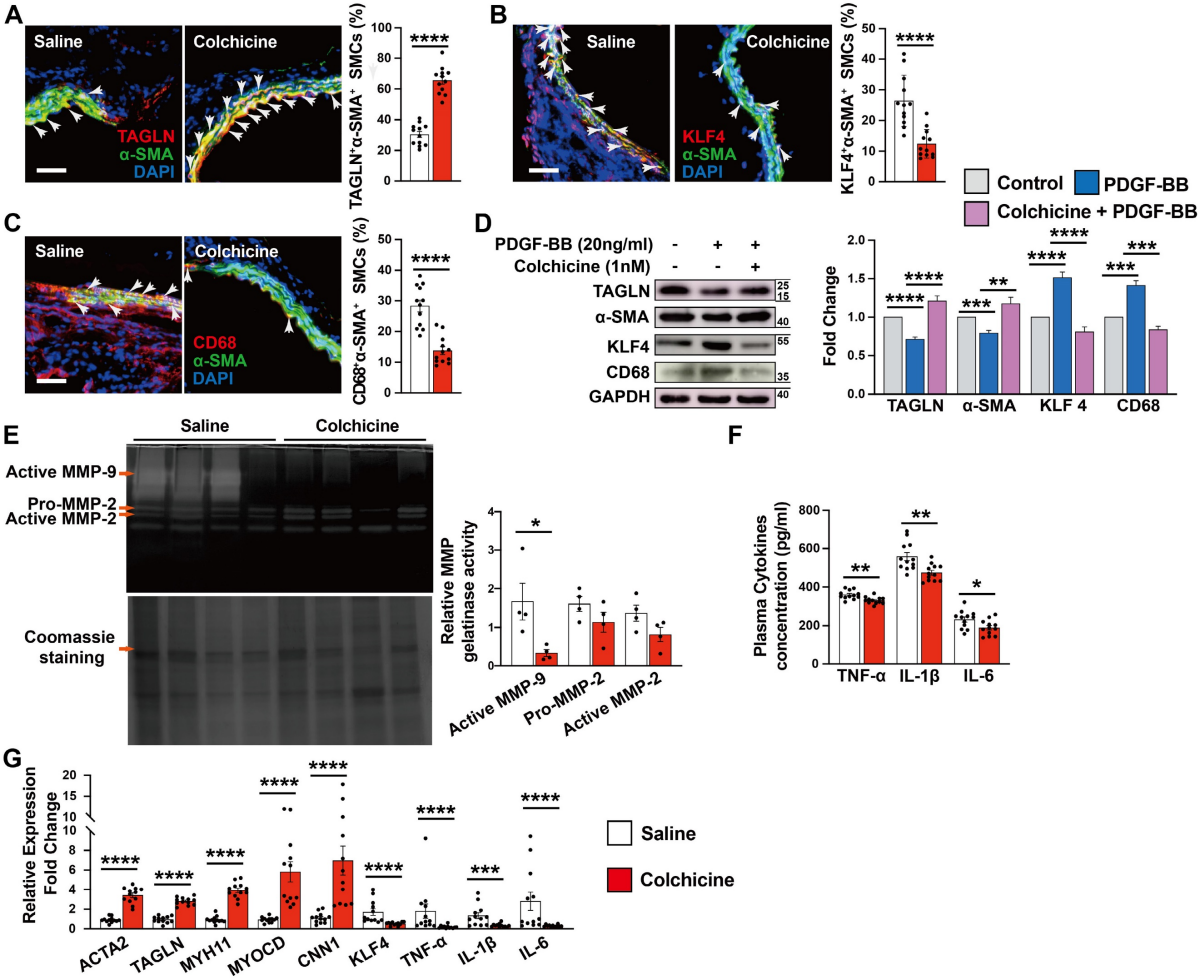
Colchicine inhibits SMC phenotypic switching in per-aortic CaPO_4_ injury-induced AAA in mice. **A)** Immunofluorescent staining of α-SMA (green) and TAGLN (red) double positive SMCs in AAA lesions. Arrows indicate α-SMA and TAGLN double positive SMCs. **B)** Immunofluorescent staining of α-SMA (green) and KLF4 (red) double positive SMCs in AAA lesions. Arrows indicate α-SMA and KLF4 double positive SMCs. **C)** Immunofluorescent staining of α-SMA (green) and CD68 (red) double positive SMCs in AAA lesions. Arrows indicate α-SMA and CD68 double positive SMCs. Scale in **A-C**: 100 μm. **D)** Human aortic SMCs were treated with PDGF-BB (20 ng/ml) with or without colchicine (1 nM) for 24 hours and harvested for immunoblot analysis of TAGLN, α-SMA, KLF4, CD68 and GAPDH (n=4). **E)** Gelatin gel zymography analysis of AAA lesions.** F)** ELISA analysis of plasma TNF-α, IL-1β and IL-6 levels. **G)** RT-PCR analysis of lesion ACTA2, TAGLN, MYOCD, MYH11, CNN1, KLF4, MMP2, MMP9, IL-6 TNF-α and IL-1β. Data are mean ± SEM, n=12 per group. *P<0.05, **P<0.01, ***P<0.001, ****P<0.0001, Student's* t* test (**A, B, C, E, F** and** G**) or two-way ANOVA followed by Bonferroni post hoc test (**D**).

Vascular SMCs display a high degree of heterogeneity and plasticity. Vascular SMC phenotype switching from a contractile to synthetic or macrophage-like phenotype plays a critical role in aortic wall stability and AAA development 29, 30. KLF4 not only plays a crucial role in modulating SMC phenotype switching, but also in endothelial function 31, 32. Immunofluorescent double staining showed that the expression of SMC contractile and early differentiation marker TAGLN in α-SMA-positive SMCs increased significantly in AAA lesions from colchicine-treated mice (**Fig. 3A**), whereas KLF4^+^α-SMA^+^ and CD68^+^α-SMA^+^ double-positive SMCs were decreased in AAA lesions from these mice (**Fig. 3B-C**). Consistent with lower expressed VCAM-1 in ECs, KLF4 expression was much higher in AAA lesions from colchicine-treated mice than that from saline-treated mice (**Fig. S2B**). In cultured human aortic SMCs, we obtained same results. Colchicine treatment markedly increased the expression of TAGLN and α-SMA in human aortic SMCs in response to PDGF-BB stimulation, but reduced the expression of KLF4 and CD68 (**Fig. 3D**). Synthetic SMCs produce matrix-degrading MMPs and pro-inflammatory cytokines that contribute importantly to AAA development and rupture 33. Gelatin gel zymography showed that the use of colchicine blunted the expression of active MMP-9, although colchicine did not affect MMP2 expression or activity (**Fig. 3E**). ELISA analysis also revealed significantly lower plasma TNF-α, IL-1β and IL-6 levels in colchicine-treated mice than those in saline-treated mice (**Fig. 3F**). Consistent with these observations, RT-PCR analysis demonstrated increased expression of SMC contractile markers ACTA2, TAGLN, MYH11, MYOCD and CNN1, but decreased expression of SMC synthetic markers KLF4, TNF-α, IL-1β and IL-6 in AAA lesions from colchicine-treated mice (**Fig. 3G**), although there were no differences in the expression of genes responsible for neutrophil adhesion (e.g. P-selectin and L-selectin) (**Fig. S6**). We detected similar phenotypic changes of vascular SMCs in Ang-II-induced AAA lesions from colchicine-treated *Apoe^-/-^* mice compared with saline-treated control *Apoe^-/-^* mice, including increase of lesion TAGLN^+^α-SMA^+^ SMCs and KLF4 expression in ECs, decrease of lesion KLF4^+^α-SMA^+^ and CD68^+^α-SMA^+^ SMCs, reduction of plasma inflammatory cytokines (TNF-α, IL-1β, IL-6), increases of lesion expression of ACTA2, TAGLN, MYH11, and CNN1, and inhibition of lesion expression of KLF4, MMP2, MMP9, IL-6, TNF-α, and IL-1β (**Fig. S2D and Fig. S7**).

Increased expression of vascular SMC contractile makers may also result from reduced vascular SMC apoptosis (**Fig. 1F and Fig. S1H**). To test this possibility, we examined the expression of SMC contractile and apoptotic makers in *Apoe^-/-^* mouse AAA lesion at 7 days after Ang-II infusion, a time point when vascular SMC phenotypic changes occur earlier than the apoptosis but before aneurysm formation 30, 34. Immunofluorescent double staining showed that colchicine helped maintain TAGLN^+^α-SMA^+^ double positive SMC contents, but decreased lesion KLF4^+^α-SMA^+^ and CD68^+^α-SMA^+^ SMC contents, although colchicine treatment did not affect lesion TUNEL^+^α-SMA^+^ SMC content, body weight gain, and systolic and diastolic blood pressures (**Fig. S8**). Together, these results suggest that attenuated synthetic SMC phenotype switching may account for the protective effect of colchicine on AAA formation.

### SOST-mediated WNT/β-catenin signaling contributes to colchicine-inhibited SMC phenotypic switching and AAA development

**Figure 4:**
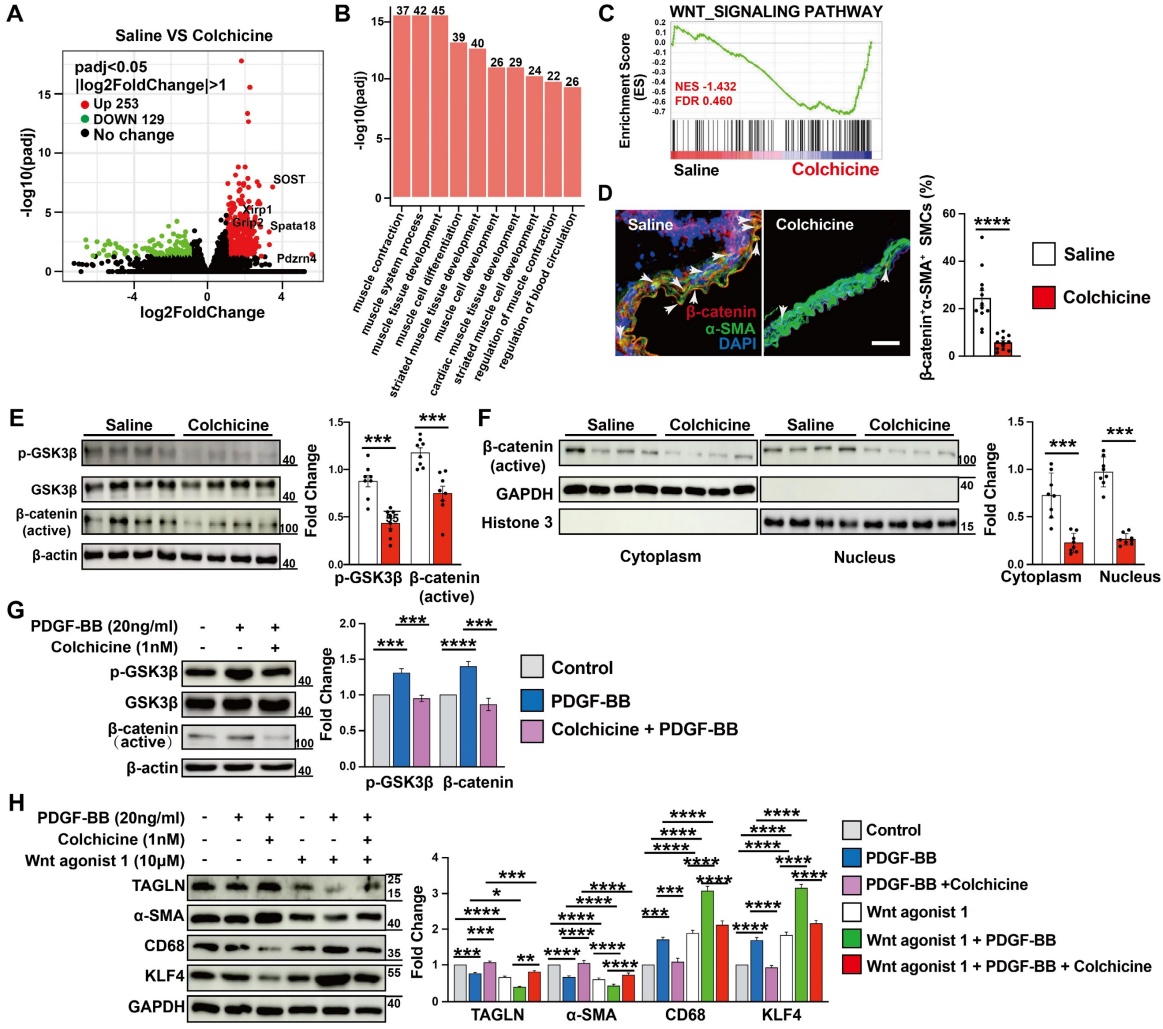
AAA lesion gene expression and SMC activation in Ang II-infused *Apoe^-/-^* mice and peri-aortic CaPO_4_-injured mice received saline or colchicine treatment. **A)** Volcano plot depicted the differentially expressed genes in AAA lesions from Ang II-infused *Apoe^-/-^* mice (n=4/each). **B)** Top enriched Gene Ontology (GO) biological process terms in Ang II-infused AAA lesions. Individual GO terms were sorted by adjusted *P* values. **C)** Gene Set Enrichment Analysis (GSEA) showed WNT signaling pathway in Ang II-infused AAA lesions. **D)** Immunofluorescent staining of media α-SMA (green) and β-catenin (red) double positive SMCs in CaPO_4_ injury-induced AAA lesions. Scale: 100 μm. Arrows indicate β-catenin accumulation in media α-SMA-positive SMC nuclear, n=12 per group. **E)** Immunoblot analysis of p-GSK3β and β-catenin (active) expression in CaPO_4_ injury-induced AAA lesions, n=8 per group. **F)** Immunoblot detection of nuclear β-catenin in CaPO_4_ injury-induced AAA lesions, n=8 per group. **G)** Human aortic SMCs were treated with PDGF-BB (20 ng/ml) with or without colchicine (1 nM) for 24 hours and harvested for immunoblot analysis of p-GSK3β and β-catenin, n=4. **H)** Human aortic SMCs were treated with PDGF-BB (20 ng/ml) with or without colchicine (1 nM) or WNT agonist (10 μM) for 24 hours and harvested for immunoblot analysis of SOST, β-catenin, p-GSK3β, GSK3β, TAGLN, α-SMA, KLF4 and CD68, n=4. Data are mean ± SEM. *P<0.05, **P<0.01, ***P<0.001, ****P<0.0001, Student's* t* test (**D-F**) or two-way ANOVA followed by Bonferroni post hoc test (**G** and** H**).

To study the mechanisms by which colchicine inhibits SMC contractile to synthetic phenotype switching and AAA development, we performed RNA next-generation sequencing (RNA-seq) in Ang-II infusion-induced AAA lesions from saline- or colchicine-treated *Apoe^-/-^* mice. We identified 382 differentially expressed genes (DEGs) in AAA lesions, with 253 up-regulated and 129 down-regulated genes (FDR < 0.05 and log_2_fold-change > 1) (**Fig. 4A**). Gene ontology (GO) analysis of these upregulated DEGs revealed that the top 10 biological processes included muscle cell contraction, differentiation, and development (**Fig. 4B**). Gene set enrichment analysis (GSEA) revealed that the WNT signaling pathway was markedly affected among the top Kyoto Encyclopedia of Genes and Genomes (KEGG) canonical pathways (**Fig. 4C**). WNT signaling plays critical roles in SMC differentiation 35, 36 and was inactivated upon colchicine treatment (**Fig. 4C**). We confirmed this observation by immunofluorescent staining and immunoblot analysis. The results showed that colchicine treatment significantly reduced β-catenin accumulation in α-SMA-positive SMCs in AAA lesions from C57BL/6 and *Apoe^-/-^* mice (**Fig. 4D and Fig. S9**), and reduced GSK-3β phosphorylation and β-catenin nuclear translocation in AAA lesions from peri-aortic CaPO_4_-injured mice and in cultured human aortic SMCs in response to PDGF-BB stimulation (**Fig. 4E-G**). SMCs exposure to PDGF-BB stimulation exhibit increased KLF4 expression and phenotypic dedifferentiation from a contractile phenotype to a synthetic or macrophage-like phenotype 37. Immunoblot analysis showed that PDGF-BB increased the WNT signaling activity and KLF4 and CD68 expression in cultured human aortic SMCs, but decreased TAGLN and α-SMA expression. Colchicine reversed these changes (**Fig. 4H**). WNT agonist 1, a WNT signaling pathway agonist 38, abolished the protective effects of colchicine (**Fig. 4H**).

**Figure 5:**
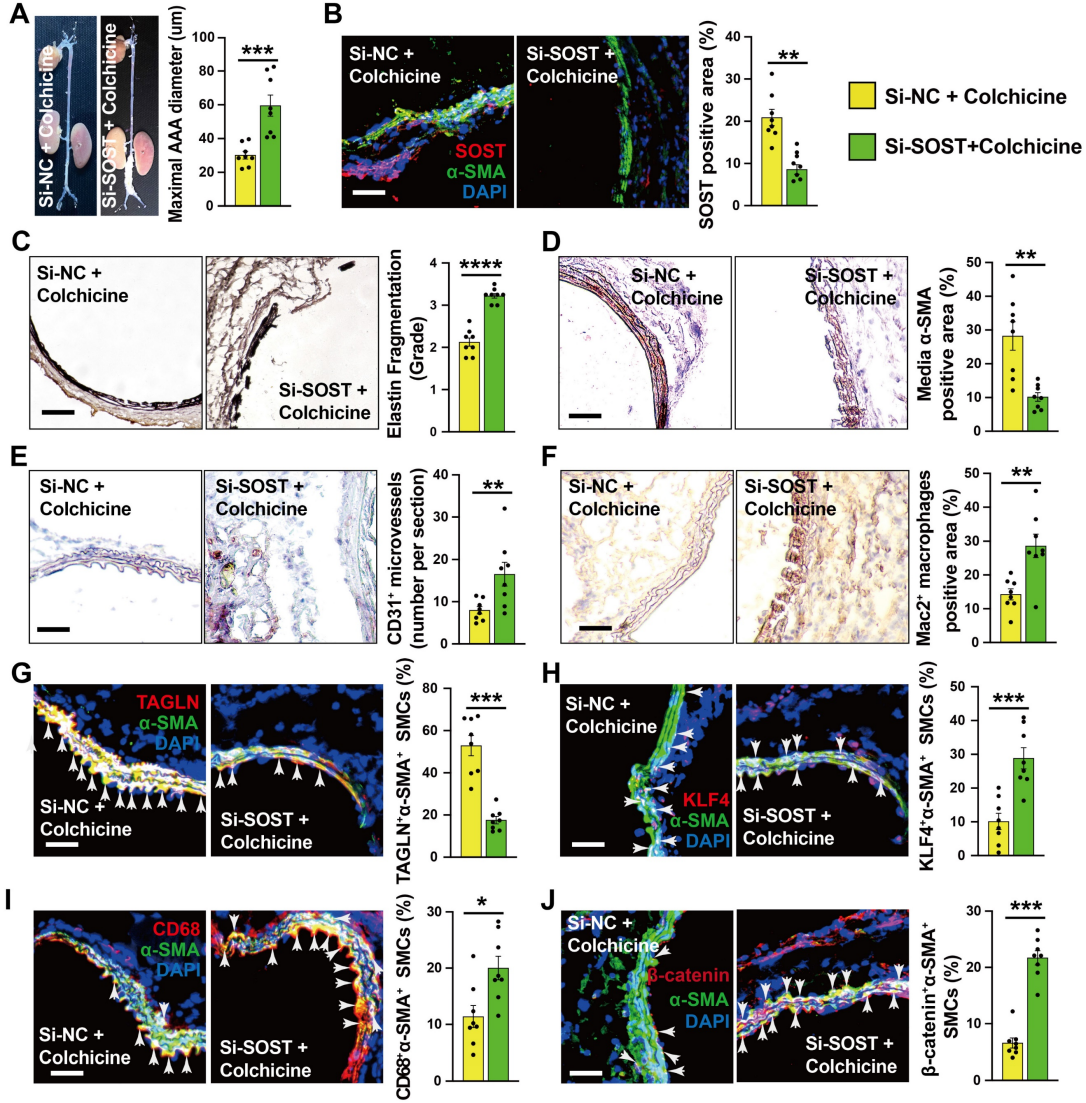
SOST Silencing abrogates the protective effects of colchicine on peri-aortic CaPO_4_ injury-induced AAA. **A)** Abdominal aortic diameters. **B)** Immunofluorescent staining of α-SMA (green) and SOST (red) to detect lesion SOST expression. **C)** Elastin fragmentation grade. **D)** Lesion α-SMA^+^ SMCs positive area. **E)** Lesion CD31^+^ microvessel numbers. **F)** Lesion Mac2^+^ macrophages-positive area. **G-J)** Immunofluorescent staining detected media α-SMA (green) and TAGLN (red) double positive (**G**), α-SMA (green) and KLF4 (red) double positive (**H**), α-SMA (green) and CD68 (red) double positive (**I**), and α-SMA (green) and β-catenin (red) double positive (**J**) SMCs. Arrows indicate media double positive SMCs. Representative images are shown to the left. Scale: 100 µm. Data are mean ± SEM, n=8 per group. *P<0.05, **P<0.01, ***P<0.001, ****P<0.0001, Student's* t* test.

To explore the underlying mechanism by which colchicine regulates WNT signaling activation, we focused on the DEGs identified by RNA-seq. Among the top up-regulated genes (**Fig. 4A**), sclerostin (SOST) is a well-known WNT signaling pathway inhibitor that is expressed highly in vascular SMCs of the abdominal aorta 39-42. Immunoblot analysis showed that colchicine significantly increased SOST expression in CaPO_4_ injury-induced AAA lesions from C57BL/6 mice and in human aortic SMCs upon PDGF-BB exposure (**Fig. S10A-B**). Immunofluorescent double staining confirmed colchicine-induced SOST expression in α-actin-positive SMCs from CaPO_4_ injury-induced AAA lesions, but not in the adventitial cells (**Fig. S10C**), suggesting that colchicine-mediated protective effects depend on SOST expression. To test this hypothesis, we mixed SOST siRNA or non-specific (NS) with pluronic F-127 gel and transplanted these mixtures to the abdominal aortas of mice that underwent peri-aortic CaPO_4_ injury-induced AAA and treated these mice with colchicine. At 7 days after surgery, RT-PCR analysis showed a 68% reduction in SOST expression in AAA lesions from SOST siRNA-treated mice compared to that from NS-siRNA-treated mice (**Fig. S11**). Moreover, SOST silencing significantly increased AAA diameter and lesion size compared with those embraced with NS-siRNA (**Fig. 5A**). Immunostaining showed that SOST knockdown promoted AAA lesion elastica fragmentation and SMC loss, and increased lesion microvessel contents and macrophage accumulation (**Fig. 5B-F**). Immunofluorescent staining showed that SOST silencing reduced AAA lesion SMC contractile phenotype (TAGLN^+^α-SMA^+^) and endothelial KLF4 expression, increased synthetic SMC contents (KLF4^+^α-SMA^+^, CD68^+^α-SMA^+^) (**Fig. 5G-I and Fig. S2F**), and enhanced the β-catenin accumulation in α-actin-positive SMCs (β-catenin^+^α-SMA^+^) and VCAM-1 expression in ECs (**Fig. 5J and Fig. S2E**). We obtained the similar results from cultured human aortic SMCs. SOST knockdown abrogated the protective effects of colchicine on SMC phenotypic switching and WNT pathway inactivation in cells stimulated with PDGF-BB (**Fig. S12**). Together, these results suggest that SOST-mediated WNT signaling inactivation contributes to colchicine-mediated protective effects on AAA development.

### Colchicine increases SOST expression by inhibiting METTL14-mediated m6A methylation

**Figure 6:**
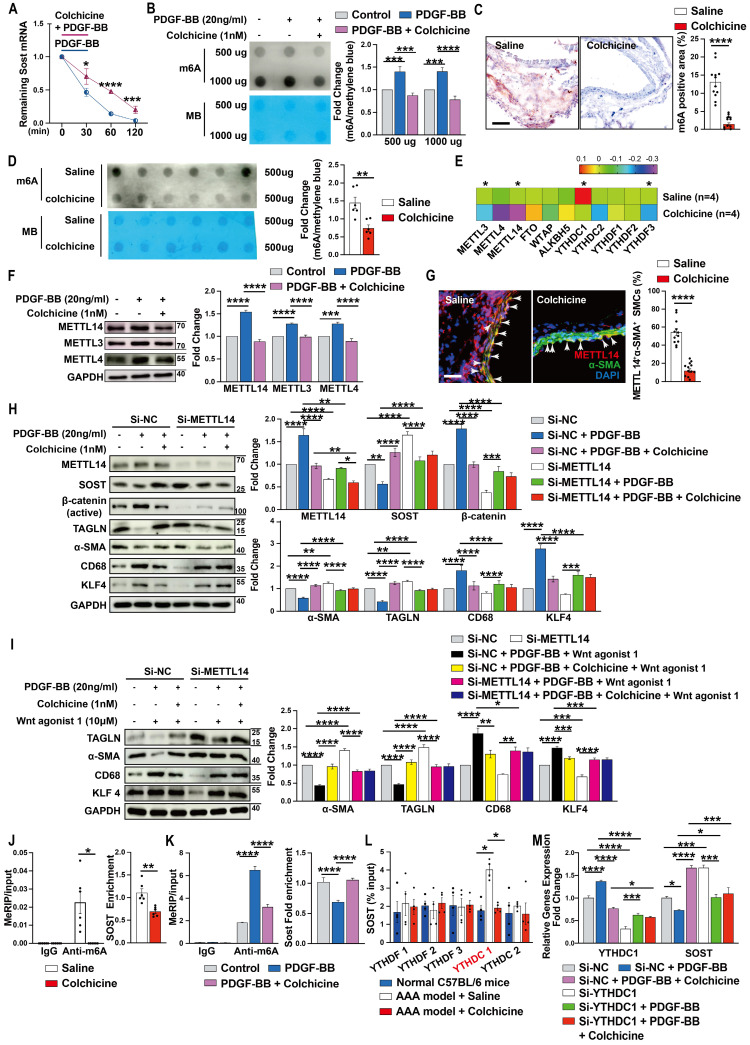
Colchicine upregulates SOST expression *via* METTL14/YTHDC1-mediated m6A hypomethylation.** A)** Colchicine increases SOST mRNA stability, n=6. **B)** Dot blot analysis of m6A methylation in human aortic SMCs treated with PDGF-BB (20 ng/ml) with or without colchicine (1 nM), n=4. MB: Methylene blue staining. **C)** m6A methylation levels in CaPO_4_ injury-induced AAA in mice treated with saline or colchicine, n=12 per group.** D)** Dot blot analysis of m6A methylation in AAA lesions from **C**. MB: Methylene blue staining. **E)** RNA-Seq detected the expression of m6A writers, erasers and readers in Ang-II infused-induced AAA lesions in mice treated with saline or colchicine, n=4 per group.** F)** Immunoblots of METTL14, METTL3 and METTL4 in human aortic SMCs stimulated with PDGF-BB (20 ng/ml) with or without colchicine (1 nM), n=4. **G)** Immunofluorescent staining of METTL14 (red) and α-SMA (green) in CaPO_4_ injury-induced AAA lesions from mice treated with saline or colchicine. Scale: 100μm. Arrows indicate α-SMA and METTL14 double positive cells, n=12 per group. **H)** Human aortic SMCs were transfected with 100 nM METTL14 siRNA (Si-SOST) or control siRNA (Si-NC) for 24 hours then treated with PDGF-BB (20 ng/ml) with or without colchicine (1 nM) for another 24 hours and harvested for immunoblot analysis of METTL14, SOST, β-catenin (active), TAGLN, α-SMA, KLF4 and CD68, n=4. **I)** Human aortic SMCs were transfected with 100 nM METTL14 siRNA (Si-SOST) or control siRNA (Si-NC) for 24 hours then treated with PDGF-BB (20 ng/ml) with WNT agonist 1 (10 μM) or with colchicine (1 nM) and WNT agonist 1 for another 24 hours and harvested for immunoblot analysis of TAGLN, α-SMA, KLF4 and CD68, n=4. **J)** RT-PCR analysis of SOST in CaPO_4_ injury-induced AAA lesions from saline- and colchicine-treated mice after MeRIP assays, n=6 per group**. K)** RT-PCR analysis of SOST in human aortic SMCs treated with PDGF-BB (20 ng/ml) with or without colchicine (1 nM) after MeRIP assays, n=6.** L)** RT-PCR analysis of SOST in CaPO_4_ injury-induced AAA lesions from saline- and colchicine-treated mice after RIP assays, n=4 per group**. M)** Human aortic SMCs were transfected with 100 nM YTHDC1 siRNA (Si-YTHDC1) or control siRNA (Si-NC) for 24 hours then treated with PDGF-BB (20 ng/ml) with or without colchicine (1 nM) for 24 hours and harvested for RT-PCR analysis of YTHDC1 and SOST, n=4. Data are mean ± SEM. *P<0.05, **P<0.01, ***P<0.001, ****P<0.0001, Student's* t* test (**A, C, D** and** G**), RStudio (**E**), or two-way ANOVA followed by Bonferroni post hoc test (**B, F, H, I, K, L** and** M**), or nonparametric Mann-Whitney *U* test (**J**).

To understand why colchicine up-regulated SOST expression in vascular SMCs, we tested whether colchicine treatment affected SOST mRNA stability. When human aortic SMCs were stimulated with PDGF-BB together with transcriptional inhibitor actinomycin D, colchicine significantly increased SOST mRNA stability (**Fig. 6A**), suggesting that colchicine regulates SOST expression at the transcription level. It is known that mRNA m6A modification controls mRNA expression and post transcription metabolism 43, 44. Dot blot of m6A using the m6A antibody showed that PDGF-BB stimulation increased global m6A methylations in human aortic SMCs. Colchicine treatment blunted this activity of PDGF-BB (**Fig. 6B**). Immunohistochemical staining and m6A dot blot assay also revealed reduced m6A methylations in AAA lesions from colchicine-treated C57BL/6 and *Apoe^-/-^* mice (**Fig. 6C-D, and Fig. S13A-B**). The results from RNA-seq also showed that METTL3 and METTL14 were remarkably decreased among the m6A writers (methylase) and erasers (demethylase) (**Fig. 6E**). Immunoblot analysis of cultured human aortic SMCs also showed that PDGF-BB increased the expression of METTL14, METTL3, and METTL4. Colchicine reduced the expression of these methyltransferases (**Fig. 6F**). Consistent with these findings, immunofluorescent double staining of lesions from peri-aortic CaPO_4_ injury-induced AAA and Ang-II infusion-induced AAA also showed that use of colchicine blunted the expression of METTL14 in lesion SMCs (**Fig. 6G** and **Fig. S13C**). In support of this hypothesis, PDGF-BB-induced changes in expression of SOST, β-catenin, α-SMA, TAGLN, CD68, and KLF4 were effectively blocked by colchicine in human aortic SMCs. siRNA-mediated knockdown of METTL14 muted the activity of colchicine (**Fig. 6H**). In the same cells, colchicine blocked the effect of WNT agonist 1-induced the changes of α-SMA, TAGLN, CD68, and KLF4 expressions. METTL14 knockdown muted these activities of colchicine (**Fig. 6I**).

To further confirm colchicine activities in decreasing m6A methylation on SOST mRNA, we used combined databases of SRAMP, RMBase, and m6A-Atlas to predict the m6A motifs 45-47 and then analyzed the MeRIP-seq dataset (GSE171371) extracted from GEO database and our MeRIP-seq dataset from patients who suffered from aortic dissection (data not shown). Use of MeRIP assays demonstrated that SOST mRNA in CaPO_4_ injury-induced mouse AAA lesions contained elevated m6A modification, which was reduced by colchicine treatment (**Fig. 6J**). Human aortic SMCs treated with colchicine yielded the same results. Colchicine effectively blocked PDGF-BB-induced SOST mRNA m6A modification (**Fig. 6K**). Use of the RNA immunoprecipitation assay, we revealed a direct interaction between SOST mRNA and the m6A reader YTHDC1 (YTH domain containing-1), but not other readers, including YTHDF1 (YTH domain family, member 1), YTHDF2, YTHDF3, and YTHDC2 (**Fig. 6L**). Knockdown of YTHDC1 increased SOST expression (**Fig. 6M**). These results indicated that colchicine increases SOST expression in a METTL14/YTHDC1-mediated m6A modification-dependent mechanism.

### AAA patients show increased m6A methylation and decreased SOST expression

**Figure 7:**
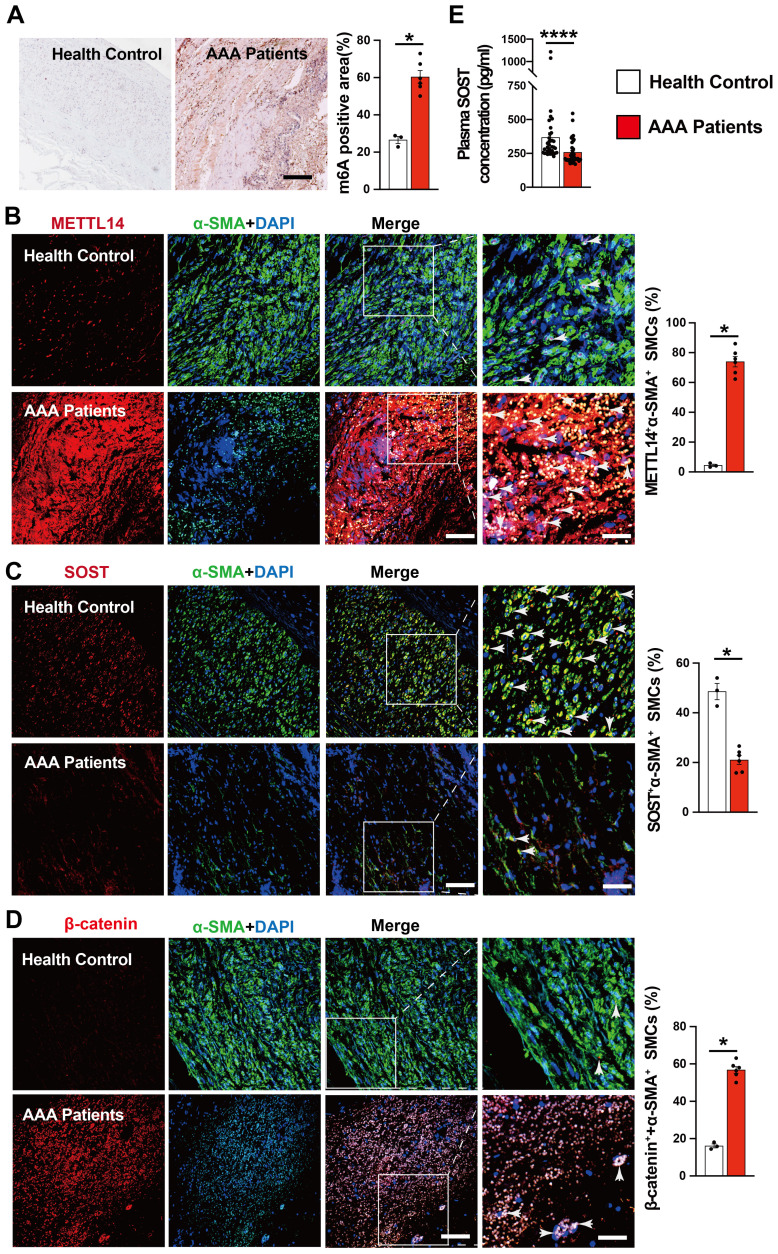
Increased m6A methylation and decreased SOST expression in human AAA lesion.** A)** m6A methylation levels in human AAA lesions and normal aorta, n=3-6 per group. **B-D)** Immunofluorescent staining detected METTL14 expression (red) in SMCs (α-SMA, green) (**B**), SOST expression (red) in SMCs (α-SMA, green) (**C**), and β-catenin expression (red) in SMCs (α-SMA, green) (**D**) from AAA lesions and normal aorta, n=3-6 per group. Scale: 100 μm, inset: 25 μm. Representative images are shown to the left.** E)** ELISA analysis of plasma SOST from AAA patients and healthy donors, n=36 per group. Data are mean ± SEM. *P<0.05, ****P<0.0001, Student's* t* test.

To explore the translational potential of colchicine in the treatment of human AAA, we examined several abdominal aortic tissues from AAA patients or healthy donors (**Table S3**). Compared with normal abdominal aortas, immunohistochemical staining showed markedly increased m6A methylation levels in AAA tissues (**Fig. 7A**). As in mouse AAA lesions, immunofluorescent double staining revealed increased numbers of METTL14-positive SMCs and β-catenin-positive SMCs, but decreased SMC SOST expression in human AAA lesions compared with those in normal human aortas (**Fig. 7B-D**). In AAA patient plasma samples, we also detected significantly lower levels of SOST by ELISA than those in sex- and age-matched control individuals without AAA (**Fig. 7E and Table S4**). These observations suggest that colchicine has protective effects on human AAA development.

## Discussion

In the current study, we demonstrated that colchicine protected peri-aortic CaPO_4_ injury- and subcutaneous Ang-II infusion-induced murine AAA development by inhibiting vascular SMC phenotype switching, SMC apoptosis, and vascular inflammation. We also identified that colchicine inhibited METTL14/YTHDC1-mediated SOST mRNA m6A modification, leading to increased SOST expression and mRNA stability, which ultimately resulted in WNT/β-catenin signaling inactivation and reduced vascular SMC synthetic phenotypic modulation. Importantly, human AAA lesions also showed increased m6A methylation and decreased SOST expression. Together, our results uncover a novel mechanism of colchicine in reducing AAA growth by inactivating the METTL14/SOST/WNT/β-catenin pathway and maintaining aortic SMC homeostasis in the aortic wall (**Fig. S14**).

Controversial observations were existed in the effective of colchicine on AAA treatment 18-20. Studies reported that daily administration of colchicine did not significantly limit AAA growth 20. Yet, evidence form us and those from others suggested a therapeutic role of colchicine on AAA 18, 19. Although we do not have exact explanation for these conflicting results, differences in AAA models and the time point of colchicine administration may account for these observations. Different from the peri-aortic CaPO_4_ injury- and Ang-II infusion-induced AAA that we used in this study, AAA lesions from combination of oral 3-aminopropionitrile (BAPN) administration and peri-aortic elastase application showed persistent aneurysm growth and more advanced-staged AAA with considerable thrombus formation and rupture, as well as higher levels of pro-inflammatory cytokines 1, 20, 48. When colchicine was given at late stage when AAA was already established, colchicine lost its inhibitory role 2, 20. In contrast, when colchicine was given at the first day of BAPN and elastase application combination-induced AAA, the same drug showed a potent a potent limitation of AAA growth 18. We and others further demonstrated that early use of colchicine prevented AAA development and rupture in several AAA models, for example, Ang-II infusion, calcium chloride and calcium phosphate application 19. Given that colchicine is a low-cost, widely available oral drug with well-known anti-inflammatory activity emerging as a promising therapeutic agent in cardiovascular disease treatment 12, 17, we believe that colchicine holds a great therapeutic potential in future human AAA treatment.

Vascular SMC phenotypic switching from a contractile differentiation phenotype to synthetic dedifferentiation phenotype in response to environmental changes contributes importantly to the pathogenesis of AAA 6, 7, 34, 49. Our observations from colchicine-treated mice and human aortic SMCs demonstrated that colchicine helped maintain the vascular SMC contractile phenotype. Because colchicine did not promote aortic SMC proliferation, one possibility is whether the protective effect of colchicine in reducing aortic SMC loss was due to reduced SMC apoptosis. Prior studies showed that vascular SMCs undergo phenotypic modulation before cell apoptosis and occur before aneurysm formation in an elastase perfusion-induced AAA model 34. In Ang II-induced AAA from our study, we demonstrated that colchicine inhibited vascular SMC dedifferentiation throughout AAA development. Macrophage-like synthetic SMCs express highly KLF4 and produce MMPs, chemokines, and pro-inflammatory cytokines, leading to SMC apoptosis, ECM degradation, and leukocyte recruitment and activation, resulting in aneurysmal dilation and rupture 31, 33, 49-51. All these changes in AAA lesion were suppressed in colchicine-treated mice, suggesting that vascular SMC phenotype switching is an early trigger of AAA development, a hypothesis that merits detailed investigation. Notably, KLF4 plays a diverse role in controlling endothelial function and vascular SMCs activities 32. Decreased KLF4 expression in ECs promoted VCAM-1 expression, thereby facilitating inflammatory cell accumulation in the vessel 52. The results from our study showed opposite KLF4 expressions in vascular SMCs and ECs upon colchicine treatment, indicating cell-type specific responses of colchicine.

Another critical question is how colchicine modulates vascular SMC phenotype switching and whether this involves direct interactions between colchicine and SMCs. Colchicine preferentially accumulates in neutrophils, livers and kidneys and directly influences those cells or organs, indicating that the actions of colchicine are cell type- and organ-specific 12, 13, 27, 53. Colchicine also inhibits myeloid cell activation by increasing hepatocyte secretion of anti-inflammatory hepatokine GDF15 13, supporting an indirect mechanism. In the current study, we showed that vascular SMCs in AAA lesions but not those in healthy vessels, or neutrophils, livers and kidneys responded to low-dose of colchicine (0.2 mg/kg/day). Therefore, colchicine directly acted on vascular SMCs in the context of AAA. Colchicine also suppressed the expression of adhesion molecules and pro-inflammatory cytokines in AAA lesions, indicating that colchicine acts on lesion ECs and macrophages 54, 55. We currently do not know whether the dose of colchicine affected its interactions with lesion ECs and macrophages.

The WNT/β-catenin pathway is activated in mouse and human AAA lesions. Inhibition of its activity represses AAA growth 39. Our study revealed a novel regulatory mechanism of colchicine in inhibiting the WNT/β-catenin pathway by increasing the expression of SOST in a METTL14-mediated m6A methylation-dependent mechanism. SOST is expressed highly in vascular SMCs, much higher than any other tested cell types in the vasculature 39-42. SOST overexpression or administration of recombinant mouse SOST prevented AAA development 39, 56. Our study also established a new role for SOST in controlling aortic SMC phenotype modulation by reducing β-catenin nuclear translocation in addition to SOST's effects on inflammation 39, 57, 58. Moreover, we showed that activation of the WNT singling using WNT agonist 1 upregulated KLF4 expression. Therefore, the WNT/β-catenin pathway controls KLF4 expression in vascular SMCs. WNT pathway activation also promotes vascular SMC proliferation and survival in atherosclerotic plaque 59-61. Yet, we found that inactivation of the WNT pathway by colchicine in SMCs exhibited a reduced apoptotic phenotype. It remains unexplained whether the role of WNT/β-catenin pathway in modulating vascular SMCs homeostasis differs under different disease conditions.

m6A modification, the most prevalent, abundant and conserved transcriptional modification, plays a critical role in controlling mRNA stability and all the cell types, including vascular SMC phenotype switching, critical to the pathogenesis of AAA 43, 44, 62-66. Here, we identified that YTHDC1, one of the downstream m6A readers, plays a distinct role in upregulating METTL14-mediated SOST expression induced by colchicine. Of note, m6A methylation level is not only elevated in vascular SMCs, but also in other cells in AAA lesion. Yet, colchicine did not increase SOST expression in those vascular cells, although a considerable reduction of m6A methylation was also observed. This probably because vascular SMCs are the main vascular cells that express SOST 39-42. These findings indicate a specific role for colchicine in modulating vascular SMC homeostasis and new insights into SOST expression regulation, although we cannot rule out other possibilities of epigenetic or post-transcriptional modifications that may also involve in colchicine-mediated SOST expression, such as histone acetylation or DNA methylation 67, 68.

Several study limitations remain in this report. Colchicine actions involve more mechanisms than what we reported in this study, such as tubulin polymerization inhibition and NLRP3 inflammasome assembly and activation 69, 70. Therefore, we cannot rule out the possibility that colchicine may prevent AAA formation using these mechanisms. Vascular SMCs display a high degree of phenotype plasticity, but this study focused only on the contractile and macrophage-like phenotypes. Other phenotypes of vascular SMCs and their roles in AAA remain untested. In this study, we used peri-aortic CaPO_4_ injury-induced AAA to study the underlying mechanisms that we identified by RNA-seq in AAA lesions from Ang-II infusion-induced *Apoe^-/-^* mice. Apparently, detailed mechanistic studies are necessary in Ang-II infusion-induced AAA model. Moreover, we used a pluronic F-127 gel embedded with siRNA to knockdown SOST expression in local infrarenal aorta. Mouse AAA model with vascular SMC-specific SOST depletion or vascular SMC-specific overexpression of METTL14 may help test the role of colchicine on SOST and vascular SMC phenotype switching in AAA development.

## Conclusion

In summary, we have demonstrated that low-dose of colchicine protected mice from AAA development by inhibiting vascular SMC phenotype switching in a METTL14/SOST/WNT/β-catenin-dependent mechanism. Colchicine also displayed potent activity in inhibiting vascular inflammation. Given the current status of AAA repair, lack of non-invasive medication contrasts the importance of trying novel, safe, and broad anti-inflammatory regimen such as colchicine. The application of colchicine in treating patients with small AAA may hold a great therapeutic potential according to the rationale of our recently launched random, double-blind, and multicenter clinical trial [Low-dose Colchicine Inhibit Abdominal Aortic Aneurysm Growth Trial (COIN), NCT05361772].

## Supplementary Material

Supplementary figures and tables.

## Abbreviations

*AAA*: abdominal aortic aneurysm
*Ang-II*: angiotensin-II
Apoe^-/-^: apolipoprotein E-deficient
*ECs*: Endothelial cells
*IL-1β*: interleukin-1β
*IL-6*: interleukin-6
*METTL14*: methyltransferase-like 14
*MMP*: matrix metalloproteinase
*PDGF-BB*: platelet-derived growth factor BB
*SMC*: smooth muscle cell
*SOST*: sclerostin
*TAGLN*: transgelin
*TNF-α*: tumor necrosis factor-α
*WT*: wild-type
*YTHDC1*: YTH domain containing-1
