# Supplementary material for: Colchicine Blocks Abdominal Aortic Aneurysm Development by Maintaining Vascular Smooth Muscle Cell Homeostasis
Source: Int J Biol Sci. 2024 Mar 17;20(6):2092–110. doi: 10.7150/ijbs.93544 (PMC11008260; doi:10.7150/ijbs.93544)
Supplement: Supplementary file 1 — Supplementary figures and tables. [file ijbsv20p2092s1.pdf]

Supplementary Materials

Colchicine Blocks Abdominal Aortic Aneurysm Development by Maintaining Vascular Smooth Muscle Cell Homeostasis

Min Chen,[1]* Dafeng Yang,[2]* Yangzhao Zhou,[2] Chongzhe Yang,[3] Wenhui Lin,[1] Jie Li,[1] Jitao Liu,[1] Jiamin Ye,[1] Yuan Liu,[1] Wenhui Huang,[1] Wentao Ma,[1] Wei Li,[4] Jiyan Chen,[1] Ying Zhang,[1] Guo-Ping Shi,[5] Jianfang Luo,[1†] Jie Li,[3†] Songyuan Luo[6, 7†]

1) Department of Cardiology, Guangdong Cardiovascular Institute, Guangdong Provincial Key Laboratory of Coronary Heart Disease Prevention, Guangdong Provincial People's Hospital, Southern Medical University, Guangzhou, China
2) Department of Cardiovascular Surgery, The Second Xiangya Hospital, Central South University, Changsha, Hunan, China
3) Department of Geriatrics, National Key Clinic Specialty, Guangzhou First People's Hospital, School of Medicine, South China University of Technology, Guangzhou, China
4) Department of Cardiology, Guangdong Provincial People's Hospital Zhuhai Hospital, Zhuhai, China
5) Department of Medicine, Brigham and Women's Hospital and Harvard Medical School, Boston, Massachusetts, USA
6) Department of Cardiology, Guangdong Cardiovascular Institute, Guangdong Provincial Key Laboratory of Hypertension, Guangdong Provincial People's Hospital, Southern Medical University, Guangzhou, China
7) Department of Cardiology, Ganzhou Hospital of Guangdong Provincial People’s Hospital, Ganzhou Municipal Hospital, Ganzhou, Jiangxi, China

*These authors contributed equally to this work.

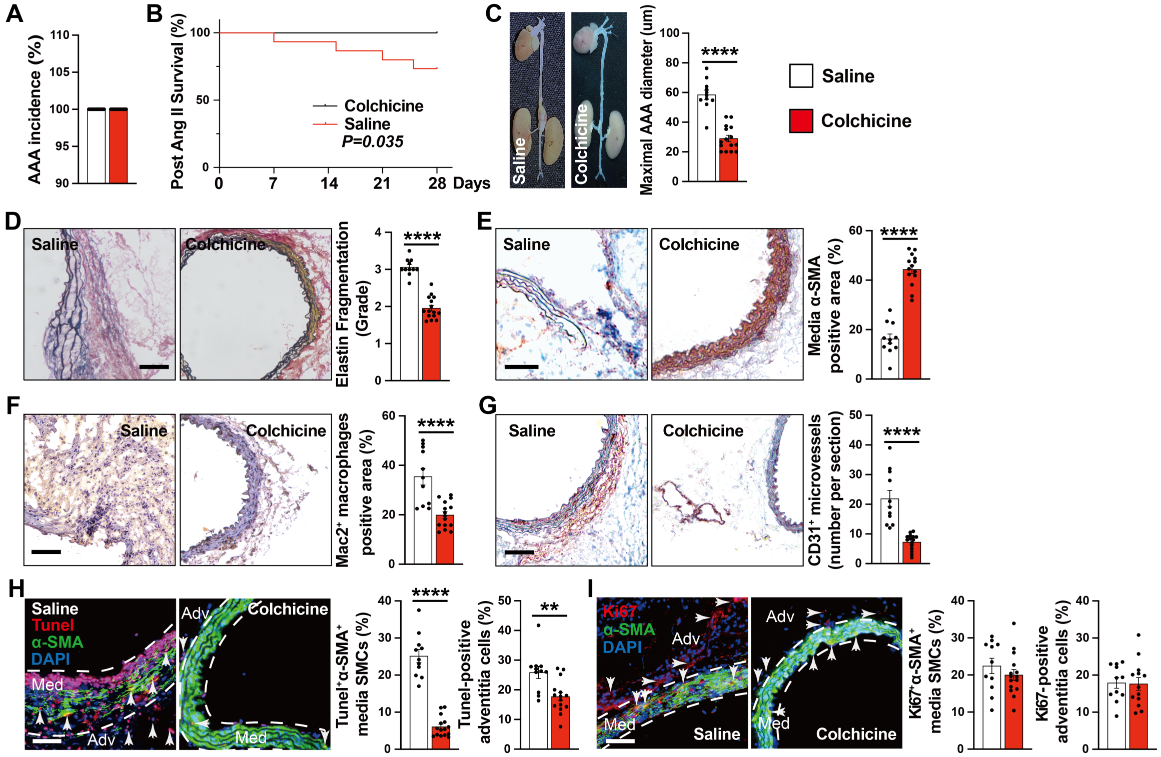

Figure S1. Colchicine inhibits Ang-II infusion-induced AAA at day 28. **A)** AAA incidence rate. **B)** Aortic rupture-associated survival rate. **C)** Aortic diameters. **D)** Elastin fragmentation grade. **E)** Lesion α-SMA$^+$ SMCs positive area. **F)** Lesion Mac2$^+$ macrophages-positive area. **G)** Lesion CD31$^+$ microvessel numbers. **H)** Immunofluorescent staining of α-SMA (green) and TUNEL (red) to detect lesion media SMC apoptosis and adventitia apoptotic cells. Arrows indicate TUNEL-positive cells. **I)** Immunofluorescent staining of α-SMA (green) and Ki67 (red) to detect lesion media proliferating SMCs and adventitial proliferating cells. Arrows indicate Ki67-positive proliferated cells. All representative images are shown to the left. Scale: 100 µm (**D-G**) and 200 µm (**H, I**). Data are mean ± SEM, n=11-15 per group. *P<0.05, **P<0.01, ***P<0.001, ****P<0.0001, χ^2 test (**A**), long-rank test (**B**), or Student's t test (**C-I**).

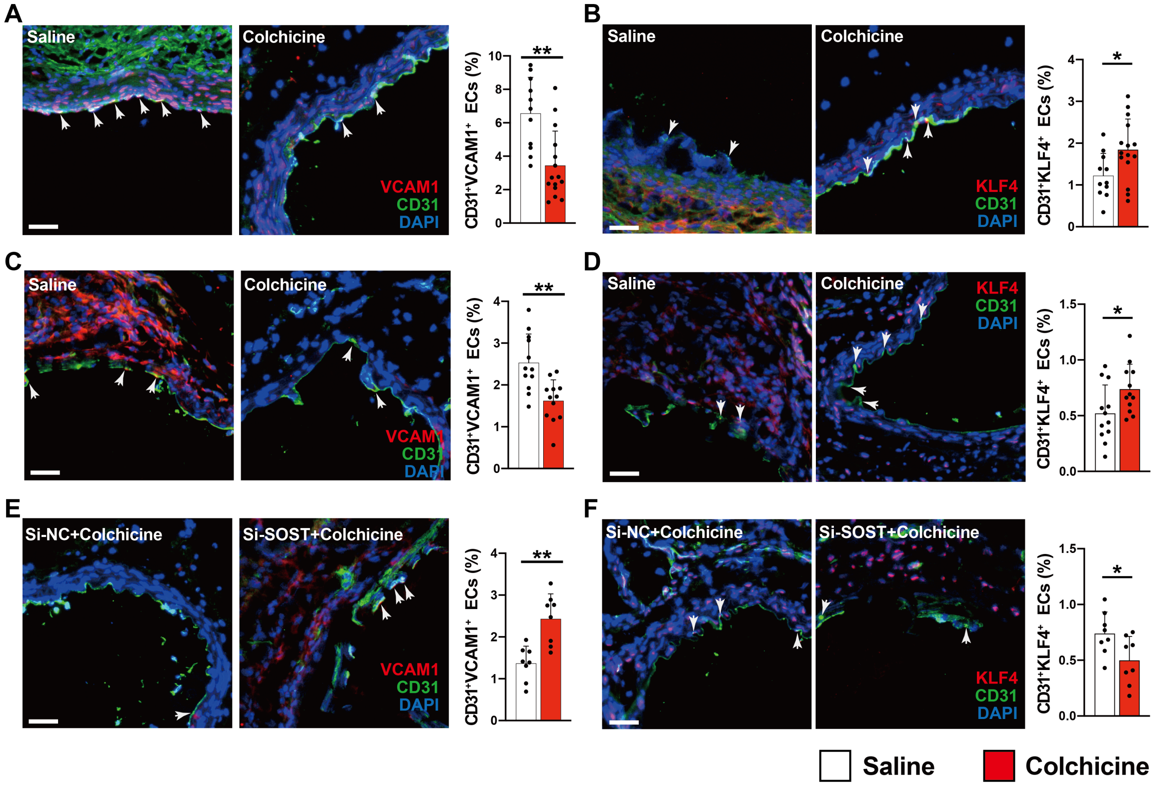

Figure S2. Colchicine increases KLF4 expression, but decreases VCAM-1 expression in endothelial cells of AAA lesion. Immunofluorescent staining of CD31 (green) and VCAM-1 (red) (**A**) or KLF4 (red) (**B**) to detect endothelial VCAM-1 or KLF4 expression in peri-aortic $CaPO_4$ injury-induced AAA lesions. Immunofluorescent staining of CD31 (green) and VCAM-1 (red) (**C**) or KLF4 (red) (**D**) to detect endothelial VCAM-1 or KLF4 expression in subcutaneous Ang-II infusion-induced AAA lesions. Immunofluorescent staining of CD31 (green) and VCAM-1 (red) (**E**) or KLF4 (red) (**F**) to detect endothelial VCAM-1 or KLF4 expression in control siRNA or si-SOST treated AAA lesions induced by peri-aortic $CaPO_4$ injury. Arrows indicate VCAM-1 or KLF4 positive endothelial cells. All representative images are shown to the left. Scale: 100 µm (**A-F**). Data are mean ± SEM, n=8-15 per group. *P<0.05, **P<0.01, ***P<0.001, Student's t test (**A-F**).

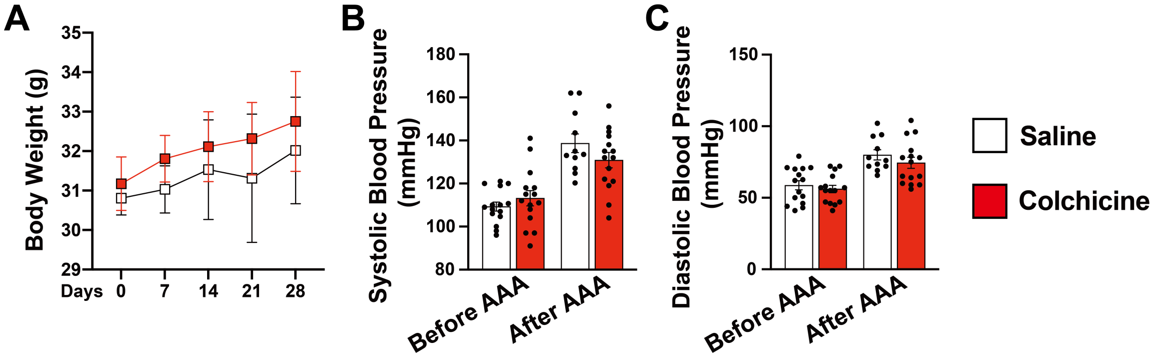

Figure S3. Colchicine does not affect body weight gain, systolic and diastolic blood pressure in Ang-II infusion mice. **A)** Body weight gain before and after AAA induction. **B)** Systolic blood pressure before and after AAA induction. **C)** Diastolic blood pressure before and after AAA induction. Data are mean ± SEM, n=11-15 per group.

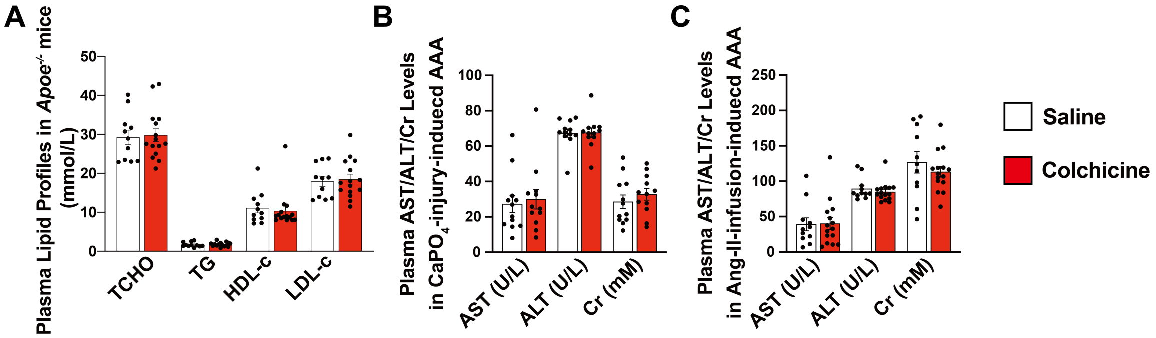

Figure S4. Colchicine does not affect lipid metabolism, liver and kidney function in $CaPO_4$-injure- and Ang-II infusion-induced AAA in mice. **A)** ELISA analysis of plasma total cholesterol (TCHO), triglyceride (TG), high-density lipoprotein cholesterol (HDL-c) and low-density lipoprotein cholesterol (LDL-c) levels in Ang-II infusion-induced AAA mice received saline or colchicine. **B/C)** ELISA analysis of plasma aspartate aminotransferase (AST), alanine aminotransferase (ALT) and creatinine (Cr) levels in $CaPO_4$ injury-induced (**B**) or Ang-II infusion-induced (**C**) AAA mice received saline or colchicine. Data are mean ± SEM, n=11-15 per group.

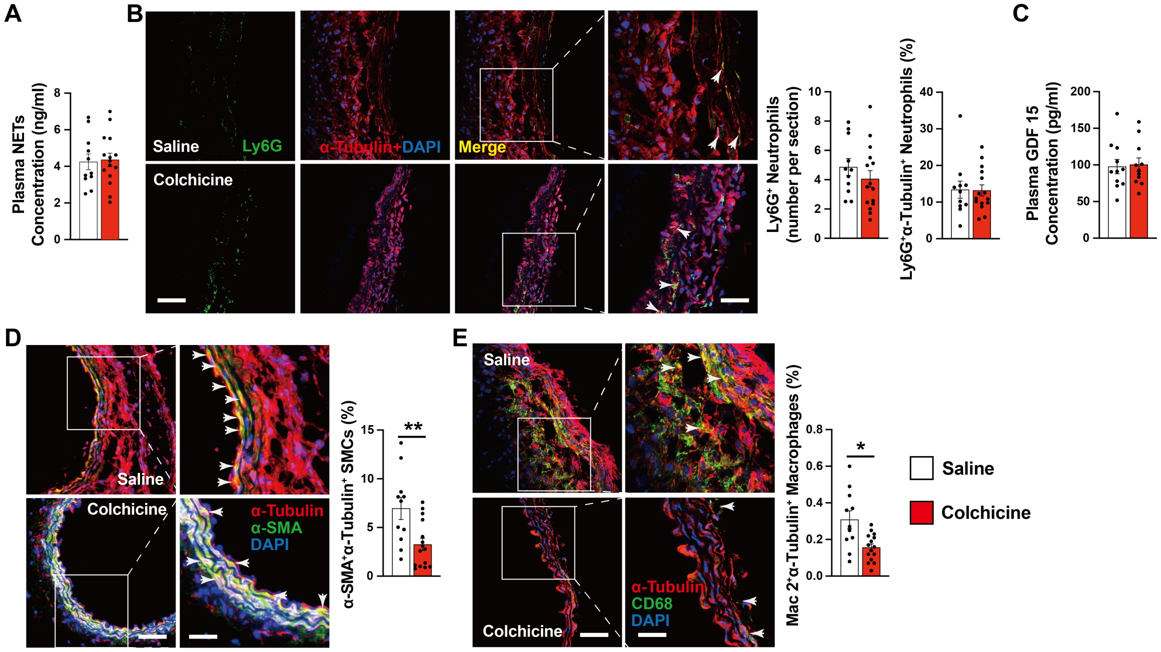

Figure S5. Colchicine does not influence neutrophil activation, but increases lesion vascular SMC tubulin depolymerization in Ang-II infusion-induced AAA mice. **A)** ELISA analysis of plasma NET levels from saline and colchicine-treated mice. **B)** Immunofluorescent staining of Ly6G (green) and α-tubulin (red) to detect lesion neutrophil accumulation and tubulin depolymerization. Arrows indicate Ly6G-positive neutrophils. **C)** ELISA analysis of plasma GDF 15 levels from saline and colchicine-treated mice. **D)** Immunofluorescent staining of α-SMA (green) and α-tubulin (red) to detect tubulin depolymerization in vascular SMCs. Arrows indicate α-SMA-positive SMCs. **E)** Immunofluorescent staining of CD68 (green) and α-tubulin (red) to detect tubulin depolymerization in macrophages. Arrows indicate CD68-positive macrophages. Scale in **B**/**D**/**E**: 100 μm, inset: 25 μm. Data are mean ± SEM, n=11-15 mice per group. *$P<0.05$, **$P<0.01$, Student's *t* test.

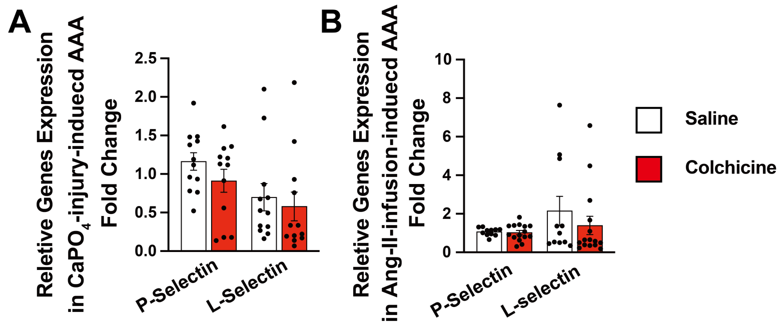

Figure S6. Colchicine does not affect AAA lesion P-selectin and L-selection expression in CaPO$_4$-injury- and Ang-II infusion-induced AAA lesions. **A/B)** RT-PCR analysis of AAA lesion P-selectin and L-selectin expression from CaPO$_4$-injury-induced AAA (**A**) or Ang-II infusion-induced AAA (**B**) in mice treated with saline or colchicine. Data are mean ± SEM, n=11-15 mice per group.

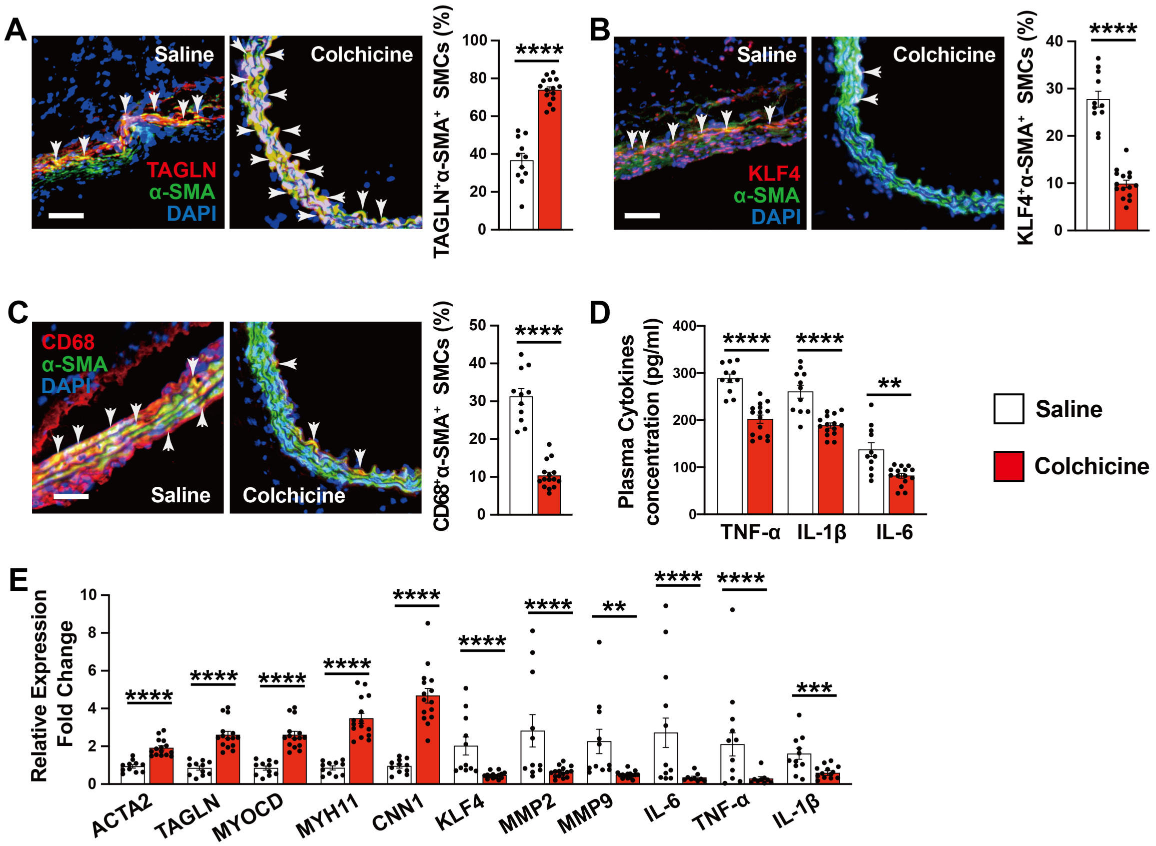

Figure S7. Colchicine inhibits lesion SMC phenotypic switching in Ang-II infusion-induced mouse AAA lesions. **A)** Immunofluorescent staining of media α-SMA (green) and TAGLN (red) double positive SMCs (arrows). **B)** Immunofluorescent staining of media α-SMA (green) and KLF4 (red) double positive SMCs (arrows). **C)** Immunofluorescent staining of media α-SMA (green) and CD68 (red) double positive SMCs (arrows). Representative images are shown to the left (**A-C**), Scale: 100 µm. **D)** ELISA analysis of plasma TNF-α, IL-1β and IL-6 from saline and colchicine-treated mice. **E)** Real-time PCR analysis of lesion ACTA2, TAGLN, MYOCD, MYH11, CNN1, KLF4, MMP2, MMP9, IL-6 TNF-α and IL-1β from saline and colchicine-treated mice. Data are mean ± SEM, n=11-15 per group. **P<0.01, ***P<0.001, ****P<0.0001, Student's t test.

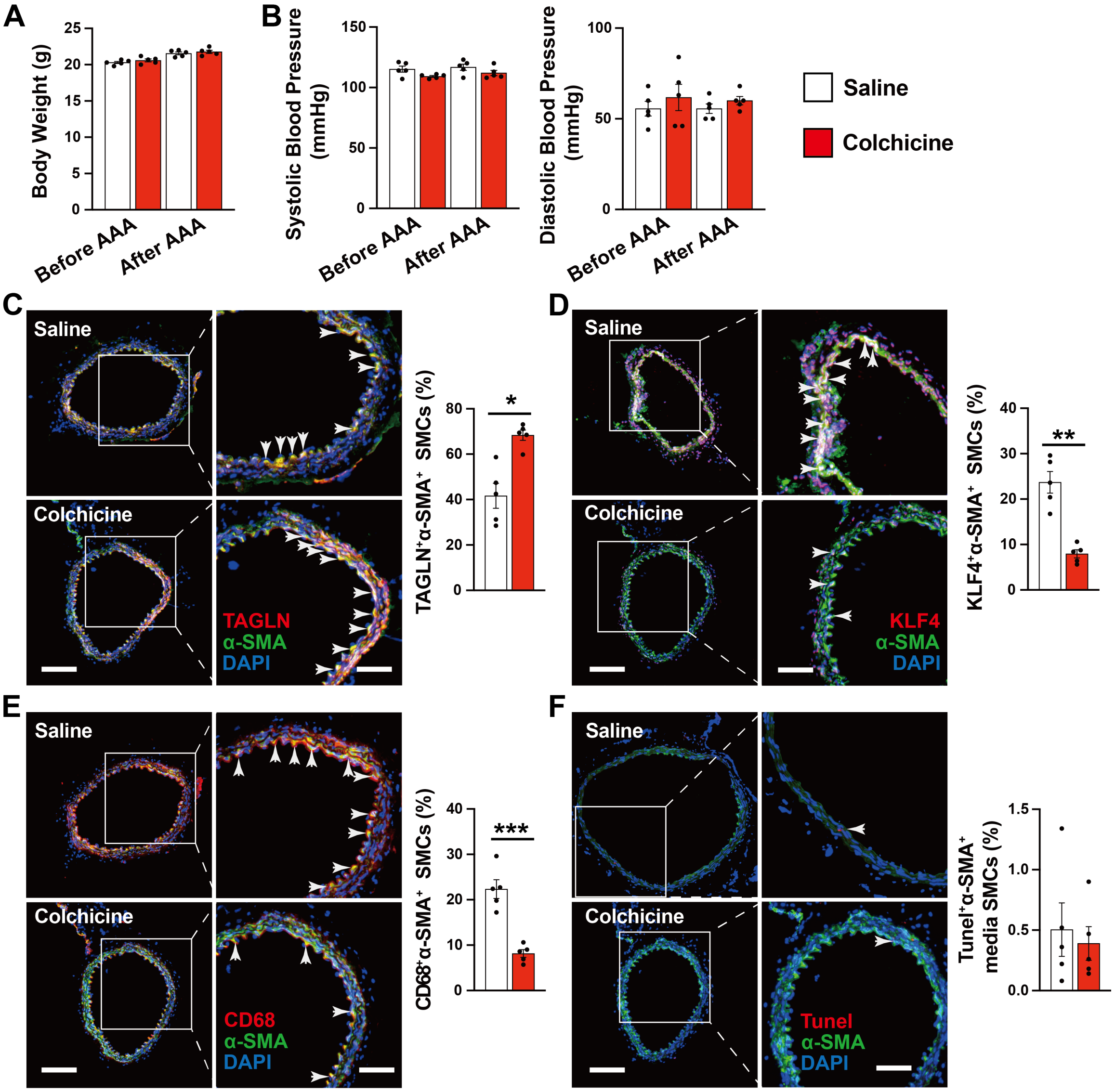

Figure S8. Colchicine maintains lesion SMC homeostasis before SMC apoptosis in Ang-II infusion-induced AAA mice at day 7. **A)** Body weight gain before and after AAA induction. **B)** Systolic and diastolic blood pressure before and after AAA induction. **C-F)** Immunofluorescent staining of media α-SMA (green) and TAGLN (red) double positive SMCs (arrows) (**C**), α-SMA (green) and KLF4 (red) double positive SMCs (arrows) (**D**), α-SMA (green) and CD68 (red) double positive SMCs (arrows) (**E**), and α-SMA (green) and TUNEL (red) double positive SMCs (arrows) (**F**). Representative images are shown to the left (**C-F**). Scale: 200 μm. Inset: 100 μm. Data are mean ± SEM, n=5 mice per group. *P<0.05, **P<0.01, ***P<0.001, Student's t test.

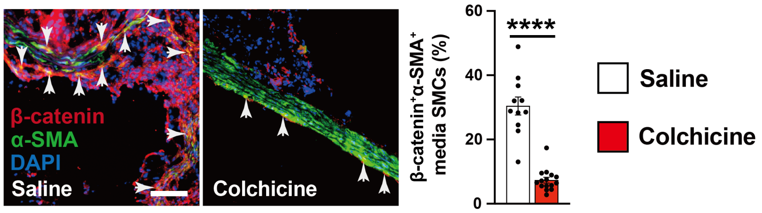

Figure S9. Colchicine inhibits β-catenin nuclear translocation in lesion SMCs from Ang-II infusion-induced AAA. Immunofluorescent staining of media α-SMA (green) and β-catenin (red) double positive SMCs. Representative images are shown to the left. Arrows indicate β-catenin accumulated in the nuclear of media α-SMA-positive SMCs. Scale: 100 µm. Data are mean ± SEM, n=11-15 mice per group. ****P<0.0001, Student's t test.

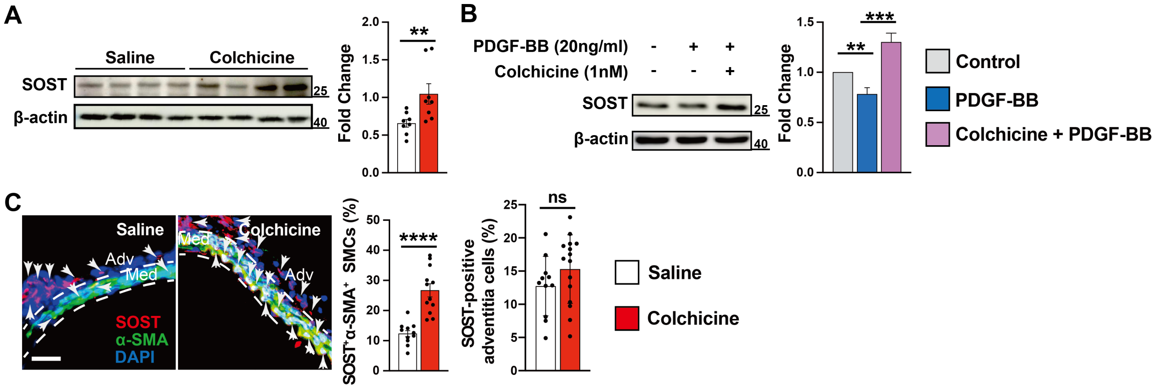

Figure S10. Colchicine increases SOST expression in SMCs. **A)** Immunoblot analysis of SOST in AAA lesions from saline and colchicine-treated peri-aortic $CaPO_4$-injured mice, n=8 per group. **B)** Human aortic SMCs were treated with PDGF-BB (20 ng/ml) with or without colchicine (1 nM) for 24 hours and harvested for immunoblot analysis of SOST, n=4. **C)** Immunofluorescent staining of α-SMA (green) and SOST (red) in AAA lesions from saline- and colchicine-treated $CaPO_4$-injured mice. Scale: 100 µm. Arrows indicate α-SMA positive SMCs, n=12 per group. Data are mean ± SEM. **P<0.01, ***P<0.001, ****P<0.0001, Student's t test (**A** and **C**) or two-way ANOVA followed by Bonferroni post hoc test (**B**).

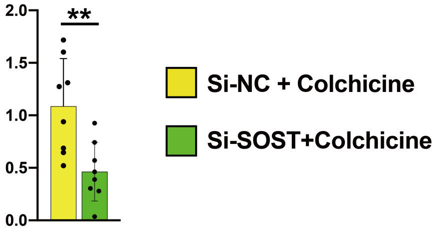

Figure S11. The efficiency of SOST knockdown in pluronic F-127 gel transplanted AAA lesions. RT-PCR analysis of lesion SOST expression. Data are mean ± SEM, n=8 per group. **P<0.01, Student's t test.

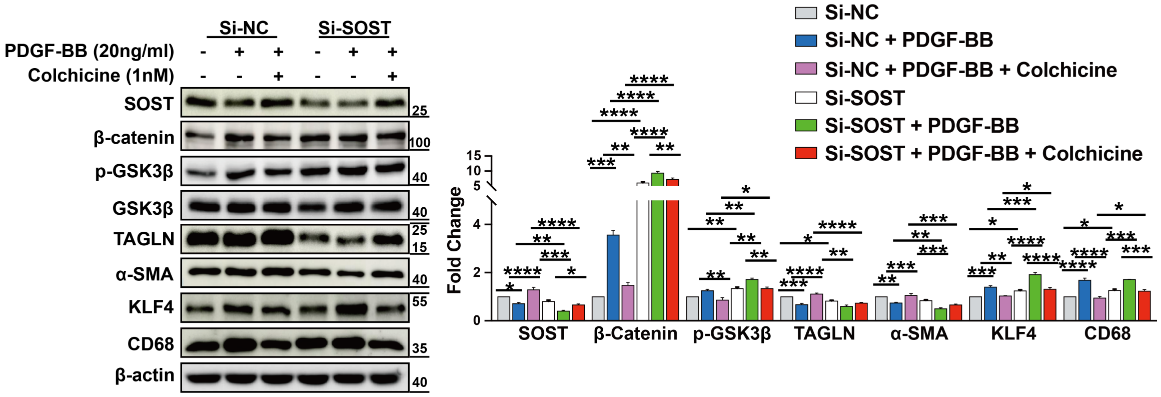

Figure S12. Knockdown of SOST abrogates the protective effects of colchicine on cultured human aortic SMCs in response to PDGF-BB stimulation-induced phenotypic switching. Human aortic SMCs were transfected with 100 nM of SOST siRNA (Si-SOST) or control siRNA (Si-NC) for 24 hours then treated with PDGF-BB (20 ng/ml) or PDGF-BB together with colchicine (1 nM) for another 24 hours and harvested for immunoblot analysis of SOST, β-catenin, p-GSK3β, GSK3β, TAGLN, α-SMA, KLF4, and CD68. Data are mean ± SEM, n=4. **P*<0.05, ***P*<0.01, ****P*<0.001, *****P*<0.0001, two-way ANOVA followed by Bonferroni post hoc test.

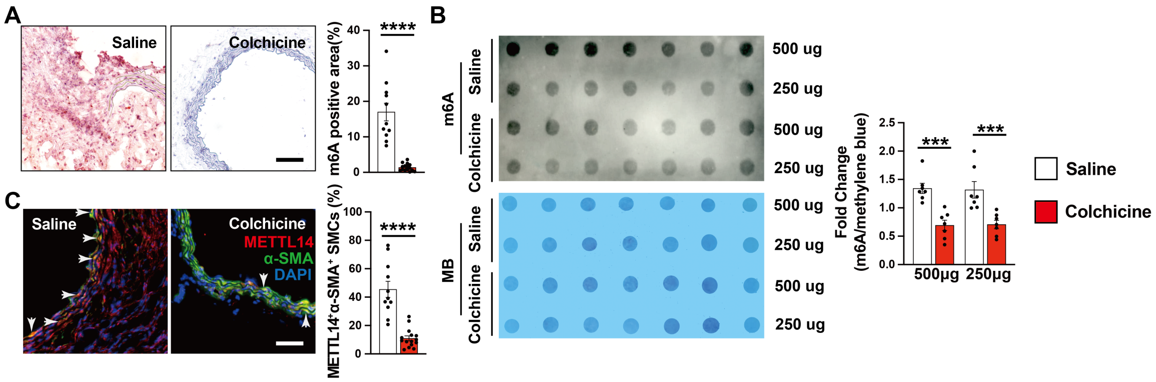

Figure S13. Colchicine inhibits AAA lesion m6A methylation in Ang-II infusion-induced AAA in mice. **A)** Immunostaining of AAA lesion m6A methylation. Scale: 100µm. **B)** Dot blot analysis of m6A methylation in AAA lesions. MB, Methylene blue staining. **C)** Immunofluorescent staining of α-SMA (green) and METTL14 (red) to detect METTL14 expression in lesion media SMCs. Scale: 100µm. Arrows indicate α-SMA and METTL14 double positive cells. Data are mean ± SEM, n=11-15 mice per group. ****P*<0.001, *****P*<0.0001, Student's *t* test.

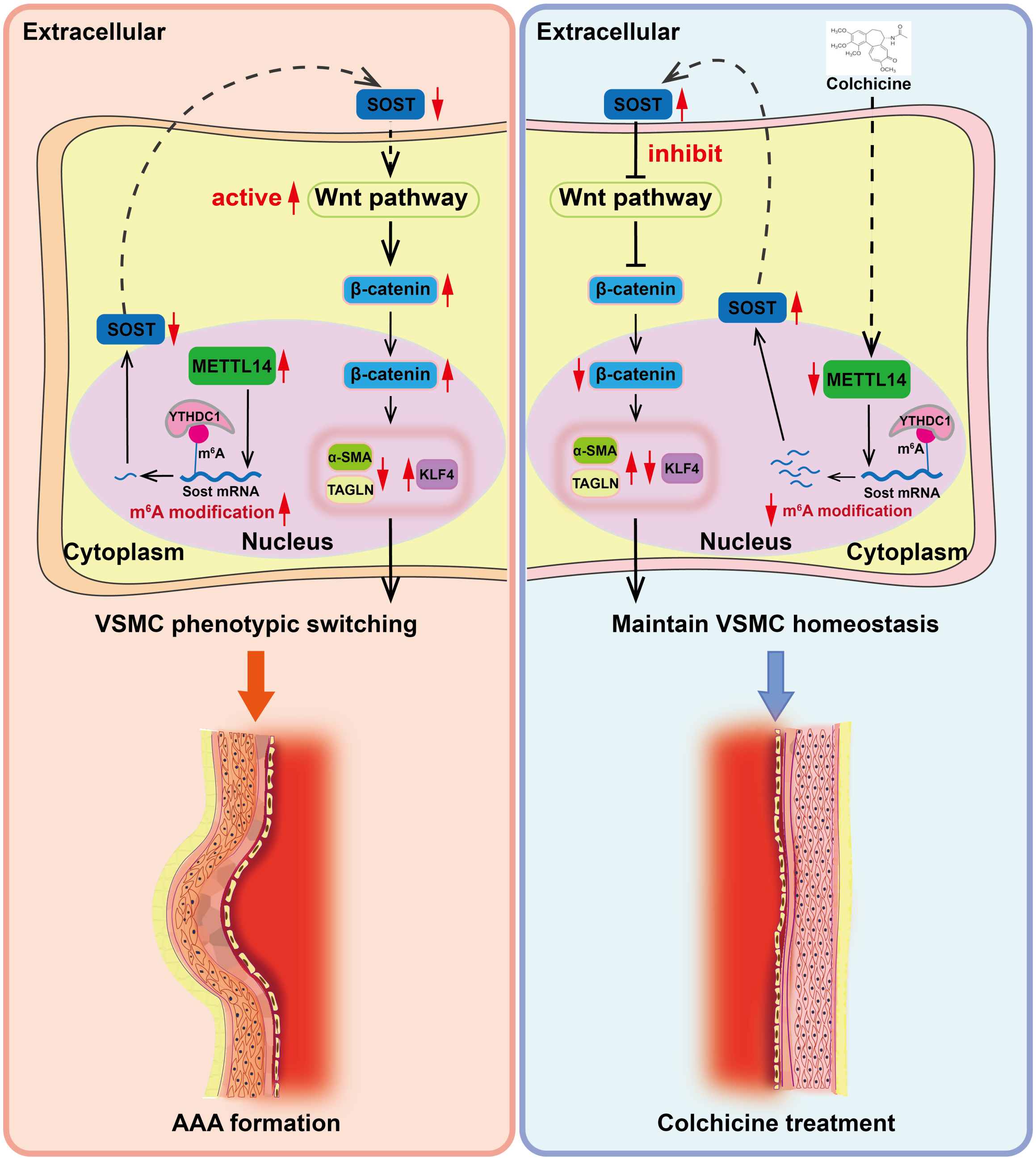

Figure S14. Proposed mechanism of colchicine activity in preventing AAA development via maintaining vascular SMC homeostasis.

Supplementary Table S1. Primers for MeRIP qPCR.

Gene name	Forward	Reverse
Human-SOST	CACACAGCCTTCCGTGTAGT	ACTCGGACACGTCTTTGGTC
Mmu-SOST	AGCCTTCAGGAATGATGCCAC	CTTTGGCGTCATAGGGATGGT

Supplementary Table S2. Primers for RT-PCR.

Gene name	Forward	Reverse
Human-SOST	ACACAGCCTTCCGTGTAGTG	GGTTCATGGTCTTGTTGTTCTCC
Human-YTHDC1	AACTGGTTTCTAAGCCACTGAGC	GGAGGCACTACTTGATAGACGA
Human-GAPDH	GGAGCGAGATCCCTCCAAAAT	GGCTGTTGTCATACTTCTCATGG
Mmu-ACTA2	GTCCCAGACATCAGGGAGTAA	TCGGATACTTCAGCGTCAGGA
Mmu-CNN1	TCTGCACATTTTAACCGAGGTC	GCCAGCTTGTTCTTTACTTCAGC
Mmu-KLF4	GTGCCCCGACTAACCGTTG	GTCGTTGAACTCCTCGGTCT
Mmu-MYH11	AAGCTGCGGCTAGAGGTCA	CCCTCCCTTTGATGGCTGAG
Mmu-MYOCD	GATGGGCTCTCTCCAGATCAG	GGCTGCATCATTCTTGTCACTT
Mmu-MMP2	CAAGTTCCCCGGCGATGTC	TTCTGGTCAAGGTCACCTGTC
Mmu-MMP9	CTGGACAGCCAGACACTAAAG	CTCGCGGCAAGTCTTCAGAG
Mmu-L-Selectin	TACATTGCCCAAAAGCCCTTAT	CATCGTTCCATTTCCCAGAGTC
Mmu-P-Selectin	GAAAGGGCTGATTGTGACCCC	AGTAGTTCCGCACTGGGTACA
Mmu-TAGLN	CAACAAGGGTCCATCCTACGG	ATCTGGGCGGCCTACATCA
Mmu-TNF-α	CCCTCACACTCAGATCATCTTCT	GCTACGACGTGGGCTACAG
Mmu-IL-1β	GCAACTGTTCCTGAACTCAACT	ATCTTTTGGGGTCCGTCAACT
Mmu-IL-6	TAGTCCTTCCTACCCCAATTTCC	TTGGTCCTTAGCCACTCCTTC
Mmu-β-actin	GGCTGTATTCCCCTCCATCG	CCAGTTGGTAACAATGCCATGT

Supplementary Table S3. Baseline characteristics of healthy donor subjects and AAA patients.

	Age	Sex
Healthy donor-1	65	Female
Healthy donor-2	63	Male
Healthy donor-3	59	Male
AAA Patient-1	66	Male
AAA Patient-2	69	Male
AAA Patient-3	73	Female
AAA Patient-4	68	Male
AAA Patient-5	69	Female
AAA Patient-6	65	Male

Supplementary Table S4. Baseline characteristics of control subjects and AAA patients.

Characteristics	Control subjects (n=36)	AAA patients (n=36)	*P* value
Age, (year)	71.9±8.5	70.4±6.7	0.074
Male sex, no. (%)	30 (83.3)	29 (80.6)	0.761
Body Mass Index (kg/m^2)	24.42±6.19	23.61±2.93	0.100
SBP (mmHg)	148±14	148±15	0.970
DBP (mmHg)	85±8	85±10	0.178
Current smoking, no. (%)	16 (44.4)	18 (50.0)	0.639
Hypertension, no. (%)	20 (55.6)	25 (69.4)	0.227
Diabetes, no. (%)	8 (22.2)	4 (11.1)	0.209
PAD, no. (%)	8 (22.2)	4 (11.1)	0.209
History of stoke, no. (%)	1 (2.8)	5 (13.9)	0.09
Stain, no. (%)	11 (30.6)	14 (39.8)	0.461
Renin-angiotensin inhibitor, no. (%)	32(88.9)	29 (80.6)	0.329
Antithrombotic agent or anticoagulant, no. (%)	36 (100.0)	36 (100)	1.000
Beta blockers, no. (%)	36 (100.0)	35 (97.2)	0.317

Values are mean ± SEM or number (percentage). SBP, systolic blood pressure; DBP, diastolic blood pressure; PAD, peripheral arterial disease. Statistical analyses were performed using Student's *t* test (age, body mass index, SBP, DBP) or χ^2 test (sex, smoking and hypertension).
